# Comparative genomics reveals adaptive evolution of Asian tapeworm in switching to a new intermediate host

**DOI:** 10.1038/ncomms12845

**Published:** 2016-09-22

**Authors:** Shuai Wang, Sen Wang, Yingfeng Luo, Lihua Xiao, Xuenong Luo, Shenghan Gao, Yongxi Dou, Huangkai Zhang, Aijiang Guo, Qingshu Meng, Junling Hou, Bing Zhang, Shaohua Zhang, Meng Yang, Xuelian Meng, Hailiang Mei, Hui Li, Zilong He, Xueliang Zhu, Xinyu Tan, Xing-quan Zhu, Jun Yu, Jianping Cai, Guan Zhu, Songnian Hu, Xuepeng Cai

**Affiliations:** 1 State Key Laboratory of Veterinary Etiological Biology, Key Laboratory of Veterinary Parasitology of Gansu Province, Lanzhou Veterinary Research Institute, Chinese Academy of Agricultural Sciences, Lanzhou, Gansu 730046, China; 2 CAS Key Laboratory of Genome Sciences and Information, Beijing Institute of Genomics, Chinese Academy of Sciences, Beijing 100101, China; 3 University of Chinese Academy of Sciences, Beijing 100049, China; 4 Division of Foodborne, Waterborne and Environmental Diseases, National Center for Emerging and Zoonotic Infectious Diseases, Centers for Disease Control and Prevention, Atlanta, Georgia 30329-4018, USA; 5 Core Genomic Facility and CAS Key Laboratory of Genome Sciences & Information, Beijing Institute of Genomics, Chinese Academy of Sciences, Beijing 100101, China; 6 Department of Veterinary Pathobiology, College of Veterinary Medicine & Biomedical Sciences, Texas A&M University, College Station, Texas 77843-4467, USA

## Abstract

*Taenia saginata, Taenia solium* and *Taenia asiatica* (beef, pork and Asian tapeworms, respectively) are parasitic flatworms of major public health and food safety importance. Among them, *T. asiatica* is a newly recognized species that split from *T. saginata* via an intermediate host switch ∼1.14 Myr ago. Here we report the 169- and 168-Mb draft genomes of *T. saginata* and *T. asiatica*. Comparative analysis reveals that high rates of gene duplications and functional diversifications might have partially driven the divergence between *T. asiatica* and *T. saginata*. We observe accelerated evolutionary rates, adaptive evolutions in homeostasis regulation, tegument maintenance and lipid uptakes, and differential/specialized gene family expansions in *T. asiatica* that may favour its hepatotropism in the new intermediate host. We also identify potential targets for developing diagnostic or intervention tools against human tapeworms. These data provide new insights into the evolution of *Taenia* parasites, particularly the recent speciation of *T. asiatica*.

## Abstract (web summary)

Only one of the three *Taenia* species causing taeniasis in humans was previously sequenced. Here the authors provide draft genomes of *Taenia saginata* and *Taenia asiatica*, analyse genome evolution of all three species, and identify potential targets for developing diagnostic markers or intervention tools.

## Graphical abstract

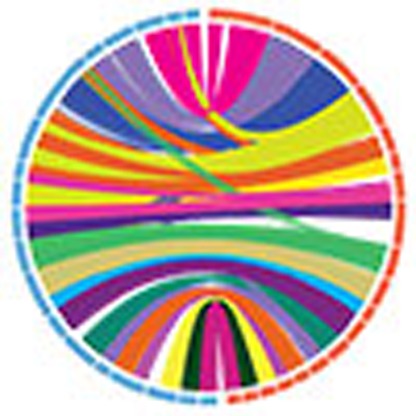

Tapeworms (cestodes) infect all major groups of animals, including humans and many economically important species. Among them, *Taenia solium* (pork), *Taenia saginata* (beef) and *Taenia asiatica* (Asian) tapeworms cause taeniasis in humans (definitive host)^1^. Pork and beef tapeworms are globally distributed, each infecting ∼50–60 million people around the world^2^,^3^. Their larvae infect swine and cattle (intermediate hosts), respectively, leading to considerable economic losses and significant burdens in global food trade^1^. Asian tapeworm is confined to only Asian endemics, and was previously confused with beef tapeworm due to their morphological similarity in adult stage until the mid-1980s when pigs were found to be the major intermediate host. It was recognized as a new species in 1993 (refs 4, 5).

Asian and beef tapeworms differ in several morphological details and predilection sites in intermediate hosts. Like *T. solium*, the cysticerci of *T. saginata* (∼10 mm in diameter) are mainly established in the bovine striated muscles, whereas those of *T. asiatica* (∼2 mm) mainly infect pig livers^6^,^7^. For *T. asiatica*, fully mature cysticerci are developed in 4 weeks (versus ∼10–12 weeks for *T. saginata*)^5^. The hepatotropic feature is considered the major reason that *T. asiatica* has been documented only in Asian countries where some populations consume raw or undercooked pork livers^8^. However, the global impact is probably underappreciated because adult Asian tapeworm can only be distinguished from beef tapeworm by molecular techniques that are not routinely used in some regions of the world, and its ability to cause human cysticercosis has not been ruled out^5^,^8^. Besides morphological similarity, earlier studies indicated that Asian and beef tapeworms share a recent common ancestor that infected hominids and bovids (resembling *T. saginata*) at the Pleistocene period in Africa, suggesting a switch of the preferred intermediate host from bovids to suids during the speciation of *T. asiatica*^9^,^10^,^11^. Their divergence date were estimated between 0.78 and 1.71 Myr ago^9^, or ∼1 Myr ago (0.24–1.64)^12^, based on substitution rates of the mitochondrial COI gene. However, it is unclear how *T. asiatica* became adapted to a new intermediate host and infection site, and evolved into a new species in a relatively short evolutionary time.

Among human intestinal tapeworms, only the pork tapeworm genome was recently reported^13^. Here we present the genomes of beef and Asian tapeworms, making the genomes of all three human taeniasis parasites available for comparative analysis to gain insights into their biological features and genome evolutions, and adaptation of *T. asiatica* to a new intermediate host. Our genome-scale analyses reveal that the divergence time between beef and Asian tapeworms coincided with the migration of *Homo erectus* from Africa to Asia. The frequent gene duplications may have contributed significantly to the speciation processes. We observe higher genome variability and more accelerated adaptive evolution in *T. asiatica*, particularly in genes involved in host–parasite interactions, physiological homeostasis and nutrient uptake. The two genomes also provide an urgently needed resource for identifying molecular targets shared by human tapeworms for developing new therapeutics, as well as species-specific genes for developing molecular diagnostic tools as described below.

## Results

### Genome features and comparison

We sequenced the *T. saginata* and *T. asiatica* genomes derived from single adult worms to ∼95-fold coverage using the Illumina platform, and assembled them into 3,626 (N50=583 kb, total size=169.1 Mb) and 6,904 (N50=342 kb, size=168.0 Mb) scaffolds with lengths >0.5 kb, respectively. (Note: for clarity, parameters will be described in order of *T. saginata* and *T. asiatica* whenever appropriate hereafter.) The draft assemblies are larger than that of *T. solium* (122.3 Mb)^13^. Both genomes have an identical 42.3% GC content, similar to those of *T. solium*, *Echinococcus multilocularis* and *E. granulosus* (41.9–43.5%)^13^,^14^, but higher than *Schistosoma* spp.^15^ and *Caenorhabditis* spp. (34.1–37.9%; Table 1; Supplementary Table 1). The completeness of the two sequenced genomes are similar, at 89.52% and 90.32%, respectively, as estimated using the Core Eukaryotic Genes Mapping Approach^16^, which are comparable with those of the two well-assembled *Echinococcus* genomes (89.11–92.74%; Supplementary Fig. 1). The two tapeworm genomes contain 10.38 and 10.90% repeated sequences, similar to other tapeworms, but lower than flukes, and all major non-coding RNA species, including conserved microRNA and transfer RNA genes (Table 1; Supplementary Tables 2 and 3).

We predicted 13,161 and 13,323 protein-coding genes in the two genomes, 77.2 and 75.7% of which were supported by RNA sequencing (RNA-seq; Supplementary Methods). More than half of these genes were mappable to the gene ontology (GO) terms (1,472 and 1,461 terms), KO identifiers in the Kyoto Encyclopedia of Genes and Genomes database (3,039 and 3,033) and Pfam domains (3,123 and 3,111). Among the predicted proteins, 2,361 (17.93%) and 2,365 (17.75%) contained transmembrane (TM) domains, and 1,094 (8.21%) and 1,048 (7.87%) possessed signal peptide sequences. These genes constitute metabolic pathways that are virtually identical to those in other tapeworms, including the loss of ability to *de novo* synthesize certain nutrients such as most amino acids, steroid hormones and lipids^13^,^14^ (Supplementary Fig. 2).

**Figure 1:**
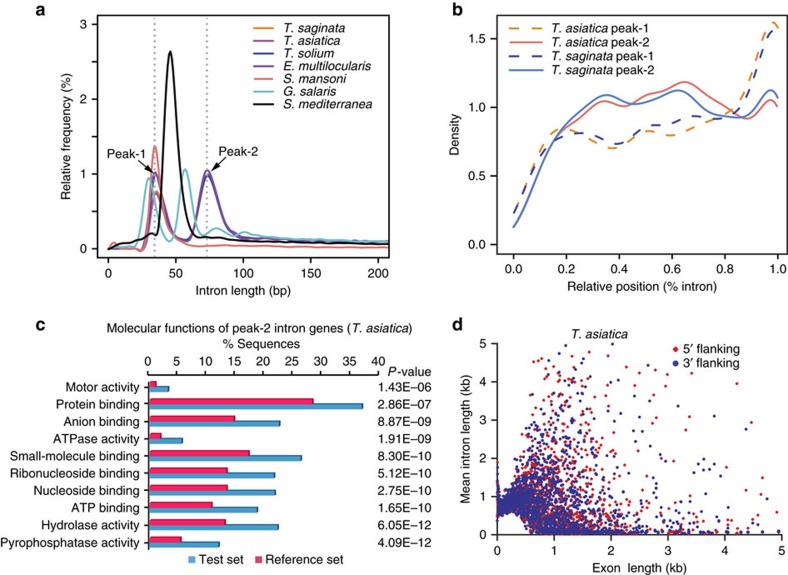
Unique intron features in the tapeworm genomes. (**a**) Bimodal length distributions of short introns in tapeworms (*T. asiatica*, *T. saginata, T. solium* and *E. multilocularis*) and monogenean *G. salaris* in comparison with the unimodal distributions in the fluke *S. mansoni* and the free-living flatworm *S. mediterranea*. (**b**) Preferential distributions of peak-1 and peak-2 introns toward 3′-end and middle regions of genes, respectively. The curves show the relationship between intron densities and relative positions from the 5′-ends of genes. (**c**) Peak-2 intron-containing genes are highly enriched to certain functional groups (shown for *T. asiatica*, by two-sided Fisher's exact test). (**d**) Length preference of introns flanking small exons (<400 bp; shown for *T. asiatica*). The minimal mean length of (5′ or 3′) introns flanking small exons that have a specific length (<400 bp) is 370 bp in *T. asiatica*.

Introns are present in 81.0 and 80.9% of the genes in the two tapeworms. The lengths of short introns follow a bimodal distribution pattern with two major peaks at ∼36 bp (peak-1 introns) and ∼73 bp (peak-2; Fig. 1a). This feature was also observed in other tapeworms^13^ and the monogenean *Gyrodactylus salaris*^17^, but not in the flukes and *Schmidtea mediterranea* (peak-1 only), suggesting the presence of this feature predating the expansion of cestodes (or before the divergence of parasitic flatworms, but lost in the fluke lineage). These peak-1 and 2 introns are located preferentially in the 3′-end and middle regions of genes, respectively (Fig. 1b). Genes containing peak-2 introns tend to possess more introns than those containing peak-1 introns (for example, average 10.52 and 14.80 introns per gene in peak-1 and 2 intron-containing sequences in *T. saginata* (*P*<0.01 by two-sided Wilcoxon-rank sum test) (Supplementary Fig. 3). Interestingly, peak-2 intron-containing genes are significantly enriched to certain functional groups (for example, pyrophosphatase activity, hydrolase activity and nucleoside binding) and cellular components (for example, cytoskeletal motor proteins and membrane proteins; Fig. 1c; Supplementary Data 1). A striking but previously unreported feature in the tapeworms is the apparent preference of the mean lengths of neighbouring introns flanking small exons. The minimal mean lengths of introns flanking small exons (<400 bp) are 502 and 370 bp long in *T. saginata* and *T. asiatica*, respectively. This feature also occurs in some other invertebrate species, for example, *S. mansoni*, *Caenorhabditis elegans* and *Drosophila melanogaster* (Fig. 1d; Supplementary Figs 4 and 5). However, the extensiveness of this feature among eukaryotes and the mechanism behind it remains to be elucidated. Introns in tapeworms are biased towards A/T bases (26.8/31.1% in *T. saginata*; 26.6/31.1% in *T. asiatica*), but not as obvious as in *S. mansoni* (31.3/33.6%) and *G. salaris* (33.8/35.3%; Supplementary Fig. 6). However, no A/T-bias in exons was observed in tapeworms, although it was found in the flukes (that is, 25.2/25.0% in *T. saginata* and 25.3/25.1% in *T. asiatica*, versus 31.5/33.2% in *S. mansoni*; Supplementary Fig. 7).

**Figure 2:**
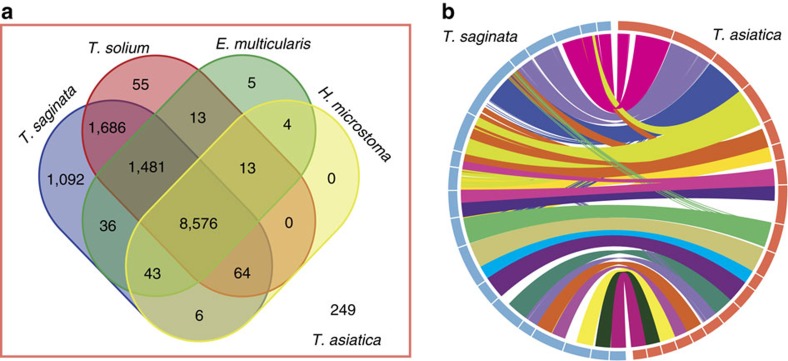
Homologues and synteny between *T. asiatica* and related tapeworms. (**a**) Homologous genes shared between *T. asiatica* and other tapeworms (that is, *T. saginata*, *T. solium*, *E. multicularis* and *H. microstoma*). (**b**) Gene block linkages between *T. asiatica* and *T. saginata*. The collinear gene blocks determined by MCScan between genome scaffolds (>1 Mb) represent 7,212 and 7,201 genes for *T. asiatica* and *T. saginata*, respectively.

We compared homologous genes among human tapeworms, and observed that 12,984 (97.5%) and 11,888 *T. asiatica* genes (90.3%) had homologues in *T. saginata* and *T. solium* (BLASTP cutoff: 1*e*^−4^; Fig. 2a). Pair-wise collinearity analysis of orthologous blocks on scaffolds revealed a higher degree of similarity between *T. saginata* and *T. asiatica* (*n*_1_=7,201, *n*_2_=7,212, 292 blocks) than between the two species and *T. solium* (*n*_1_=6,055, *n*_2_=6,058, 303 blocks; Fig. 2b). Similarly, a higher nucleotide identity (92.26%) with larger alignable blocks (total 138 Mb, mean length=5.4 kb) was observed between *T. saginata* and *T. asiatica* (versus ∼88.53% identity, ∼108 Mb total and 3.8 kb mean lengths between beef/Asian and pork tapeworms). These data agree with their taxonomic affiliations.

### Gene duplications during divergence of tapeworms

**Figure 3:**
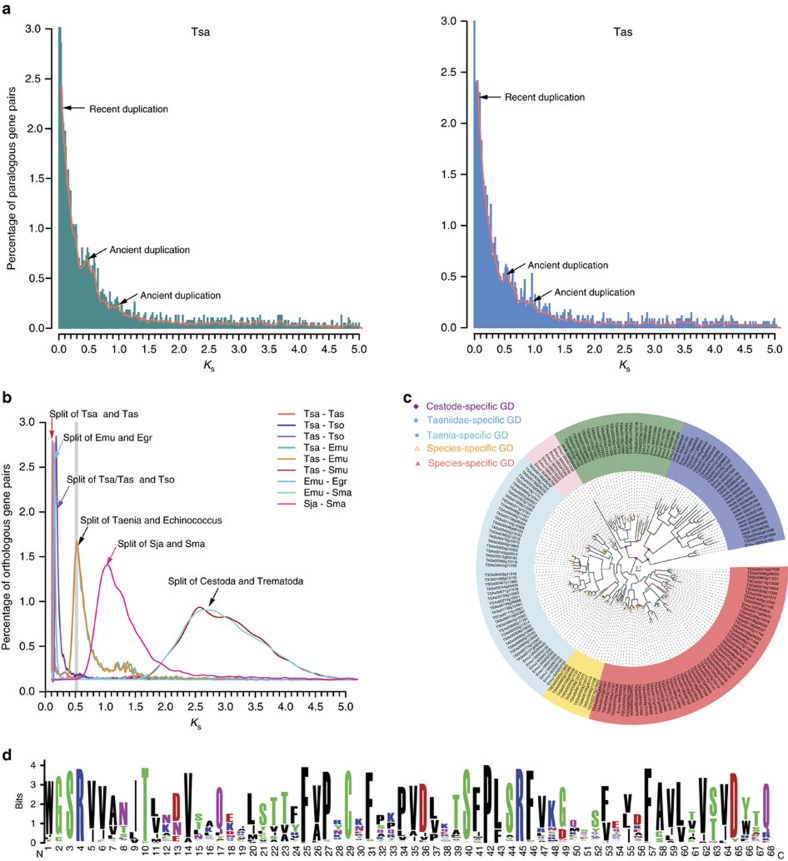
Gene duplications revealed by *K*_s_ analysis. (**a**) The percentage of paralogous gene pairs of duplicated genes along with *K*_s_ values are typically L shaped in the *T. saginata* (Tsa) and *T. asiatica* (Tas) genomes, indicating the occurrence of continuous gene duplication events and the losses of duplicated genes over the time. (**b**) Distribution of *K*_s_ values in orthologous genes with peaks indicating the splits between various flatworms *T. asiatica* (Tas), *T. saginata* (Tsa), *T. solium* (Tso), *E. granulosus* (Egr), *E. multilocularis* (Emu), *S. mansoni* (Sma) and *S. japanicum* (Sja). The grey bar indicates the divergence point between the *Taenia* and *Echinococcus* lineages. (**c**) Extensive duplications of diagnostic antigen gp50 genes in the tapeworm lineage (*Taenia*, *Echinococcus* and *Hymenolepis*). (**d**) Sequence logo shows the conserved and distinct sequence characteristics of the gp50 sequences from the tapeworms. The sequence logo was generated from 183 sequences aligned at the conserved blocks selected by Gblocks with a less stringent selection (http://molevol.cmima.csic.es/castresana/Gblocks_server.html).

Gene duplication (GD) is known as a primary source of materials for evolutionary innovations and adaptations^18^,^19^, in which the age of a GD event is proportional to the number of synonymous substitutions per synonymous site (*K*_s_) of paralogous genes. In the genomes of *T. saginata*, *T. asiatica* and other related parasitic flatworms, the *K*_s_ distributions are typically quasi-exponentially L shaped (Fig. 3a,b; Supplementary Fig. 8), which agrees well with the notions that most duplications are young (for example, 4.36 and 4.98% GDs with *K*_s_<0.01 in the *T. saginata* and *T. asiatica* genomes) because of the continuous loss of duplicated genes over the time. These events are mostly derived from small-scale gene duplications (SSGDs), predominated by dispersed duplications (for example, 71.95% in *T. saginata*, 74.14% in *T. asiatica* and 69.94% in *E. multilocularis*), followed by tandem (16.69, 15.69 and 15.08%) and proximal duplications (10.75, 8.90 and 11.29%; Supplementary Table 4) in the current assemblies.

Although retrotransposons are the major elements subjected to GDs in the *T. saginata* and *T. asiatica* genomes, several functional homologous groups (including surface antigens, HSP70, ubiquitin conjugating enzyme, ryanodine receptor 44f, cyclin-dependent kinase, puromycin-sensitive aminopeptidase and zinc-finger proteins) appear to have also experienced continuous and extensive SSGDs during the evolution history of the tapeworm lineage (Fig. 3c; Supplementary Figs 9 and 10; Supplementary Table 5). These SSGD events resulted in many overrepresented super-families with high sequence diversities accompanying the diversification of their biological functions. Among them, the frequent duplications and retentions of tapeworm-specific surface antigens (for example, Taeniidae antigens and diagnostic antigen gp50) are indicative of their importance in the parasite survival and/or adaptations to new environments. Indeed, Taeniidae antigens are known to play important roles in the evasion of host immunity^20^, while the diagnostic antigen gp50 proteins are glycosyl phosphatidylinositol-anchored membrane glycoproteins also heavily involved in interacting with the host immune system^21^. The gp50 gene family appears to have duplicated more extensively, driven mostly by duplicative transpositions and tandem duplications along the Taeniidae and *Taenia*-specific evolution history (Fig. 3c). These duplicated genes have been differentially retained in different tapeworms, and might have carried out multiple functions through neo-/sub-functionalization with greatly divergent sequences (Fig. 3d), although their precise biological roles remain to be illustrated.

As a major force in evolution, species-specific GDs can lead to the differentiation of gene functions, thus facilitating the species-specific adaptation and divergence^18^. Both *T. saginata* and *T. asiatica* genomes possess a large number of recently duplicated genes (involving 866 and 1,075 in-paralogs after their divergence, respectively) that were derived from 481 and 614 duplicate events along each lineage, respectively. The duplicate genes appear to arise at a high average rate in their genomes (0.0321 and 0.0404 duplicates per gene per Myr for *T. saginata* and *T. asiatica*, respectively), similar to those in estimated in *E. multilocularis* (0.0304) and *C. elegans* (0.0208)^22^, suggesting a probable high degree of plasticity of their genomes (Supplementary Methods, section 8.9). Species-specific GDs were also shown by gene enrichment analysis, in which differential distributions of significantly enriched gene categories in biological processes (*P*<0.05 by two-sided Fisher's exact test) were observed between *T. asiatica* (mostly enriched in nucleosome assembly, cilium movement and ribosome localization) and *T. saginata* (mostly enriched in protein glycosylation; Supplementary Table 6), indicative of nonrandom processes of gene retentions in each genome. These observations suggest that the high rate of origin of GDs and preferential retentions of duplicated gene families might have contributed, at least partially, to the divergence of the two closely related tapeworms.

Duplicated genes in the process of acquiring new functions at the time of species separation are likely to contribute to species differentiation^19^. Using out-paralogous genes from *T. solium* as outgroups, we compared the evolutionary rate for each pair of the in-paralogous genes arising after the divergence of *T. saginata* and *T. asiatica*, and identified a number of newly duplicated genes that evolved significantly asymmetrically between paralogous pairs (88/592 in *T. saginata*, 72/804 in *T. asiatica*; *P*<0.05 by Tajima's relative rate test), implying that they were prone to diverge functions. Particularly, the cytoskeleton components (for example, actin, dynein heavy chain and kinesin), tegument surface antigens (for example, EG95 and gp50), ion transporters/channels (for example, ryanodine receptors 44f, solute carrier family 12 and multidrug resistance protein) and growth/development-related proteins (for example, fibroblast growth factor receptor 4, round spermatid basic protein 1 and segment polarity protein disheveled) appear to not reach at a stable rate yet in *T. asiatica* (Supplementary Data 2). These proteins are probably important in the speciation of *T. asiatica*, considering their roles in the tegument maintenance, homeostasis regulation and growth/reproduction. The galactosyltransferase gene family may play a particularly important but yet undefined biological role in *T. saginata* because of its continuous duplications along tapeworm evolution. In addition, the evolutionary divergence of recently duplicated gene pairs is echoed by the divergences in their expression patterns (*R*^2^=0.2462 in *T. saginata*; 0.0114 in *T. asiatica*) (Supplementary Fig. 11) and gene structures (accounting for 23.82 and 15.05% in-paralogous pairs in *T. saginata* and *T. asiatica*), indicative of their possible functional divergences.

### Speciation history and accelerated evolution in *T. asiatica*

We collected 102 single-copy genes conserved in 10 flatworms and related species for estimating the divergence dates of tapeworms using a relaxed-clock Bayesian approach^23^. The genome-based analysis suggested that beef and Asian tapeworms diverged at 1.142 Myr (0.55–1.43, 95% highest probability density) in the early Pleistocene period (Supplementary Fig. 12), which was close to previously estimated date (∼1.0 Myr ago) based on mitochondrial genes^11^,^12^. This split predated the domestication of pigs and cattle (∼10,000 years ago)^24^, and the migration out of Africa of *Homo sapiens* (∼100,000 years ago)^25^, but fell within the periods of population expansion and migration of *H. erectus* from Africa to Asia (from ∼1.8 to 0.4 Myr ago)^26^. Currently, the closest relatives of *T. saginata* and *T. asiatica* (for example, *T. simbae*) are only found in Africa or adjacent regions^10^,^11^. If the ‘minimum number of host shifts' theory was assumed, our finding agrees with an earlier speculation^9^,^10^,^11^ that the common ancestor of beef and Asian tapeworms (*T. saginata* like) first colonized humans at or before early Pleistocene, and the hominid ancestor *H. erectus* acquired this tapeworm from bovids in Africa and then transmitted it to suids in Asia. We further speculate that the persistent hunting activity in *H. erectus* permitted long and consistent interactions among three host species, thus mediating the transfer of *T. saginata*-like ancestor from bovids to suids, giving rise to *T. asiatica*.

Despite high molecular and morphological similarities between Asian and beef tapeworms, the nucleotide substitution rate (branch-length) in protein-encoding genes of *T. asiatica* (0.00467 mutation per site) is 1.27-fold higher than that of *T. saginata* (0.00379 mutation per site) (Supplementary Fig. 13; Tajima's relative rate test, *P*<0.01). In addition, the nucleotide mutation rate in the *T. asiatica* genome is at 4.09 × 10^−9^ (3.27–8.52 × 10^−9^) mutations per site per year (versus ∼3.32 × 10^−9^ in *T. saginata* and ∼2.82 × 10^−9^ in *T. solium*), which is ∼10-fold higher than that of humans (0.33–0.47 × 10^−9^)^27^. The higher mutation rate provided a greater genome variation for selection and adaptation needed for the divergence/speciation of *T. asiatica* from *T. saginata*. However, the mechanism leading to the accelerated evolution rate in the *T. asiatica* genome is unclear.

By mapping useful sequence reads of short paired-end libraries to the assembled genomes, we detected substantial numbers of heterozygous single-nucleotide variations (SNVs). The overall SNV rate in *T. asiatica* is 2.97-fold higher than in *T. saginata* (Supplementary Table 7). The genomes of *T. asiatica* and *T. saginata* contained 60,734 (362 sites per Mb) and 20,700 (122 sites per Mb) high-quality heterozygous SNVs, respectively. Among them, 6.90% (*T. asiatica*) and 5.96% (*T. saginata*) were located in protein-coding genes, in which the most significantly enriched genes were transporters in *T. asiatica*, including those for ions (*n*=32), organic anions (*n*=11), amino acids (*n*=5) and sulfates (*n*=2; Supplementary Data 3). These proteins are mostly involved in maintaining cellular homeostasis and nutrients absorption (Supplementary Fig. 14). In addition, we observed more small indels (size=1–5 bp) in the *T. asiatica* genome (2,359 indels) than in *T. saginata* (1,014 indels; Supplementary Table 7). Both natural mutations over the time and genetic exchanges between individuals might contribute to heterozygosity, but their individual contributions in *T. asiatica* could not be established here due to the lack of intra-species genetic diversity data.

**Figure 4:**
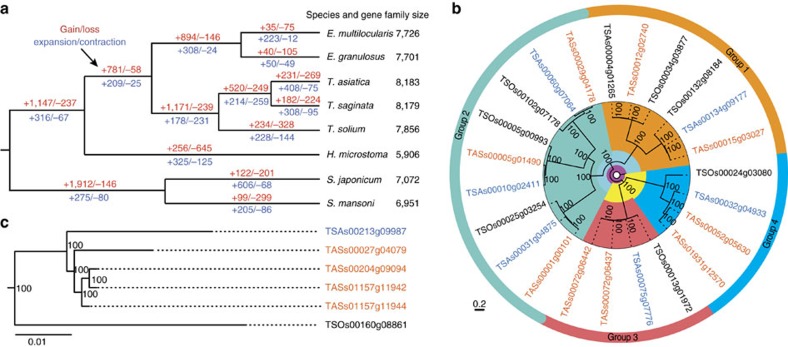
Evolution of gene families in the flatworms and roundworms. (**a**) The dynamics of gene family sizes in the genomes of *T. saginata*, *T. asiatica*, *T. solium*, *E. granulosus*, *E. multilocularis*, *H. microstoma*, *S. japonicum* and *S. mansoni*. Numbers above and below the branches indicate gene family gains/losses (red) and the expansions/contractions (blue), respectively. (**b**) Phylogenetic reconstruction clustered low-density lipoprotein receptor (LDLR) genes from *T. saginata*, *T. asiatica* and *T. solium* into four groups, in which group-4 LDLR genes were expanded only in the *T. asiatica* genome (Supplementary Methods, section 8.8). (**c**) The expansion of fatty acid desaturases (FADS) in the *T. asiatica* genome, compared with that in the *T. saginata* genome (Supplementary Methods, section 8.8).

The *T. asiatica* genome also experienced more gene family gain (*n*=231) and expansion (*n*=408) than *T. saginata* (*n*=182 and 308; Fig. 4a). The gained gene families are mainly novel domains of unknown functions, possibly related to certain specialized adaptations, while the gene copy-number variations are related to functional extension. Intriguingly, significant expansions (branch-specific *P* values<0.05) of low-density lipoprotein receptor (LDLR) and fatty acid desaturase (FADS) genes were noticed in *T. asiatica* (*n*=9 and 4 versus *n*=7 and 1 in *T. saginata* or *T. solium*; Fig. 4b,c). We speculate that this expansion played an important role in the switch of the intermediate host from cattle to swine by promoting the establishment of cysterici of Asian tapeworm in the lipid-rich liver.

### Adaptive selection in the *T. asiatica* genome

Positive selection is an important source of evolutionary innovation and one of the major forces driving species divergence. To evaluate the role of positive selection in the evolution of *T. asiatica*, we selected 1:1 orthologous genes from six tapeworms for branch-site model analysis by Phylogenetic Analysis by Maximum Likelihood (PAML)^28^, and identified 134 and 102 positively selected genes (PSGs) in the *T. asiatica* and *T. saginata* genomes (likelihood ratio test, *P*<0.05; Supplementary Data 4). We analysed the PSGs in *T. asiatica* to examine whether these rapidly evolving genes were enriched for specific functions after the divergence from *T. saginata*. We observed evolutionary pressures on some essential genes in cellular processes, including those involved in transcription, translation and regulating protein degradation (for example, various ribosomal proteins, tRNA guanine N7 methyltransferase, bifunctional aminoacyl-tRNA synthetase, small subunit processome component 20, transcription factors and ubiquitination-associated proteins; Supplementary Data 4).

Adaptive selection signals were also observed in some genes associated with specialized survival environment for *T. asiatica*, but not in *T. saginata*. For instance, selection was detected in genes involved in pH maintenance and ion homeostasis (for example, carbonic anhydrase, glutamate receptor ionotropic kainite and amiloride-sensitive cation channel 4), implying adaptation to the new host internal environment. In addition to *LDLR* and *FADS* genes, we observed PSGs involved in lipid scavenge (for example, Niemann Pick C1 protein, fatty acid-binding protein and glycolipid transfer protein) and glycolysis (for example, pyruvate kinase and fructose-2,6-bisphosphatase) that were probably beneficial to *T. asiatica* in establishing infection in the lipid/sugar-rich liver in pigs. The tegument is essential for protecting parasitic flatworms from attacks by host defence systems^29^,^30^,^31^. We observed strong selection signals in genes responsible for maintaining body surface integrity in Asian tapeworm, including cytoskeleton-associated proteins^32^,^33^ (for example, myotubularin protein, myosin heavy chain, dynein heavy and light chains, kinesin-related proteins, intraflagellar transport protein, calponin, katanin and 4.1 protein, ezrin, radixin, moesin (FERM) domain-containing protein), cell adhesion/junction (for example, tight junction protein, β-catenin protein, protocadherin gamma and FRAS1-related extracellular matrix protein), and a glycosyltransferase gene probably involved in forming the thick glycocalyx layer on the tegumental surface^13^,^34^.

### Proteins involved in host–parasite interactions

We analysed proteases, protease inhibitors (PIs) and excretory/secretory (E/S) proteins that are commonly involved in interacting with hosts and modulating host immune responses^35^,^36^. Particularly, secreted proteases can modulate host Th2 immune responses against helminths^37^. We predicted 157 and 161 proteases, plus 142 and 155 non-protease homologues, in the *T. saginata* and *T. asiatica* gnomes, which were comparable to those in *E. multilocularis*^13^ (Supplementary Data 5 and 6). They belong to five major classes (aspartic, cysteine, metallo, serine and threonine), predominated by metallo- (*n*=46–48), cysteine (*n*=41–44) and serine proteases (*n*=27–30; Supplementary Fig. 15). In addition, 23 and 26 proteases are encoded by the top 10% highly transcribed genes in larval *T. saginata* and adult *T. asiatica* (Supplementary Table 8). The two genomes encode 70–71 PIs that are all serine, cysteine and metalloprotease inhibitors, including I39 family PIs that interact with endopeptidases regardless of the catalytic type (Supplementary Data 7). The largest PI family is I02 (*n*=22, 14.0% in *T. saginata* and *n*=20, 12.4% in *T. asiatica*), which are serine PIs (aka Kunitz inhibitors; Supplementary Table 9). Several families of proteases and PIs were among the most enriched E/S proteins (Supplementary Tables 10 and 11). By comparison with PIs in *S. mansoni*, we identified several tapeworm-specific inhibitor families (for example, I87, I21 and I93), suggesting that tapeworms and flukes employ lineage-specific mechanisms to regulate protease activities.

The secretomes are large in *T. asiatica* (*n*=824, 6.18%) and *T. saginata* (*n*=885, 6.72%; Supplementary Tables 10 and 11; Supplementary Fig. 16). Many of them are proteases and PIs as described above. The two genomes also encode a large set of other classes of E/S proteins that may be involved in modulating host immune responses. For instance, some ‘Taeniidae antigens' (*n*=24 in *T. asiatica* and *n*=39 in *T. saginata*) could impair neutrophil chemotaxis and/or modulate Th2 polarization^38^. Another large E/S family is venom allergen-like proteins, which are known to modulate host immune function and regulate sexual development of parasites in the host^39^.

### Molecular targets for intervention and diagnosis

Together with the previously reported *T. solium* and *Echinococcus* genomes, the availability of *T. saginata* and *T. asiatica* genome sequences allowed us to identify potential targets shared by all human tapeworms but divergent or absent in hosts for developing therapeutics. We identified 75–78 G-protein-coupled receptors (GPCRs) and 353–355 protein kinases in *T. asiatica* and *T. saginata* that are well-known classic drug targets (Supplementary Data 8 and 9). Most GPCRs are rhodopsin family proteins (*n*=63), while protein kinases cover ∼10 major classes, in which 180 kinase groups (Supplementary Data 9) could not be clustered with those from the reference species (that is, human, *D. melanogaster* and *C. elegans*), thus may serve as potential ideal drug or vaccine targets against the parasitic helminthes. Ligand-gated ion channels (LGICs) are validated targets for many current antihelminthic drugs. We identified at least 33 members of three major LGIC families (glutamate-activated cationic channels, cys-loop LGIC and ATP-gated ion channels), and ∼20 members of related families (that is, cyclic-nucleotide-gated cation channel and amiloride-sensitive sodium channel related; Supplementary Data 10). Most of these drug targets are conserved among tapeworm genomes, thus might potentially serve as broad-spectrum drug targets.

We further searched for parasite-specific sequences from the potential drug targets and host–parasite interaction-associated proteins, and identified 34 sequences (*T. saginata*) and 45 sequences (*T. asiatica*) with no homologues in mammals (Supplementary Data 11 and 12). Most of these sequences were supported by transcription data, including several homologues of known drug targets. Among them, cystatin and phytochelatin synthase are the top drug target candidates because they are present in all tapeworms and critical in interacting with hosts and heavy-metal detoxification, respectively^13^,^40^.

The tapeworm genomes were searched for species-specific genes potentially valuable in developing molecular and/or immunological diagnostic tools, particularly those for specific detection of Asian tapeworm. We collected all single-copy genes in the *T. saginata* and *T. asiatica* genomes, and ranked them by nucleotide sequence divergence as a community resource for developing molecular detection tools (Supplementary Data 13). We also recovered 15 (versus *T. saginata*) and 110 (versus *T. solium*) high-confidence species-specific protein-coding genes in the *T. asiatica* genome (Supplementary Table 12). However, the feasibility of these genes in developing immunological assays needs experimental evaluations.

## Discussion

*T. asiatica* is morphologically indistinguishable from *T. saginata* in adult stage, but shares the same intermediate host with *T. solium*. Its speciation and switch of the intermediate host are intriguing evolutionary questions. Our comparative analysis revealed that the three human taeniasis parasites share many common genomic features but differ from each other in the evolutions and diversifications of certain specialized gene families, and reaffirmed the sister relationship between *T. asiatica* and *T. saginata*. Genome-based analysis suggests that Asian and beef tapeworms diverged ∼1.14 Myr ago, which coincides the migration of *H. erectus* to Asia, rather than the more recent migration of *H. sapiens*^9^,^11^. Thus, the speciation of *T. asiatica* predated that of *H. sapiens*. The divergence between the Asian and beef tapeworms might have been driven by the differential GDs in their genomes that display typically L-shaped distribution patterns. The high rates of extensive and continuous duplications, differential retentions and subsequent functional diversifications of gene families in the two tapeworm genome (for example, families associated with the cytoskeleton components, tegument surface antigens, ion transporters/channels in *T. asiatica* and glycosylation in *T. saginata*) might have significantly contributed to the speciation of *T. asiatica*.

We observed accelerated evolution in *T. asiatica* in mutation rate, heterozygosity and gene family gain/expansion that are all at higher rates than in *T. saginata*, suggesting that this parasite is of high evolution vigour in adaptation to new host environments. These observations challenge the hypothesis that *T. asiatica* species is at risk of extinction due to its minimal genetic diversity and limited geographical distribution^41^,^42^,^43^. Given that those studies were only based on analyses of a limited number of mitochondrial or nuclear genes, the real intra-species diversity within the *T. asiatica* populations may still need further large-scale investigations.

The PSGs in *T. asiatica* are more concentrated to gene families involved in internal homeostasis (for example, carbonic anhydrase), tegumental development (for example, cytoskeletal proteins, cell junction proteins and glycosyltransferases) and lipid uptake (for example, LDLR and fatty acid-binding protein), which are probably associated with the establishment of new immune-evasion and nutrient uptake strategies at the lipid-rich infection site (liver) in a new intermediate host (pigs). Our analysis indicates that tegument and membrane proteins in tapeworms are under particularly high evolutionary pressure in adaptation to new hosts, as evidenced by the rapidly evolved new genes/adaptive evolution/gene expansion associated with the recent host switch or speciation in *T. asiatica*. The tegument surface antigens (for example, Taeniidae antigens and diagnostic antigen gp50) may be of particular importance in tapeworm's survival and/or adaptations to new environments due to their critical roles in interacting with the host immune systems.

We identified several sets of proteins that might serve as broad-spectrum drug targets in tapeworms, including kinases, GPCRs and ion channels, as well as two proteins (cystatin and phytochelatin synthase) that could serve as potentially ideal targets in *T. saginata* and *T. asiatica*. Due to the morphological similarities, misdiagnosis between *T. saginata* and *T. asiatica* is not uncommon. Recently, several molecular approaches using sequence-specific DNA probes, PCR-based RFLP and multiplex PCR based on mitochondrial sequences were explored for differential diagnosis of the two *Taenia* tapeworms^44^,^45^,^46^. We also provide here a list of nuclear genes based on sequence divergence between the two closely related tapeworms for development of new molecular diagnostic tools.

## Methods

### Samples and preparations

Adult worms of *T. saginata* and *T. asiatica* were isolated from two patients (one worm per patient) in Yunnan Province, China. The study was approved by the ethics committee of Affiliated Hospital of Dali University (Yunnan, China) and patients have given written consent to publication of this study with the exclusion of any personal identifiers. Larvae (cysticerci) of *T. saginata* were obtained from the skeletal muscle of an experimentally infected calf 7 weeks after infection. The animal was cared in accordance with good animal practice according to the Animal Ethics Procedures and Guidelines of the People's Republic of China, and the study was approved by the Institutional Committee for the Care and Use of Experimental Animals of Lanzhou Veterinary Research Institute, Chinese Academy of Agricultural Sciences (no. LVRIAEC2010-002). Genomic DNA was extracted from freshly collected middle proglottids for constructing two paired-end libraries (300 and 500 bp inserts) and three mate-pair libraries (1, 5 and 10 kb) for *T. saginata*, and two paired-end libraries (500 bp) and seven mate-pair libraries (2, 2, 3.5, 5, 5, 7 and 10 kb) for *T. asiatica* (Supplementary Methods, section 1). Messenger RNA was isolated from *T. saginata* larvae and the middle proglotids of *T. asiatica* for the construction of paired-end cDNA libraries (300 bp). For each worm, the clonality was confirmed by the distribution of minor allele frequencies of the heterozygous sites (Supplementary Fig. 17).

### High-throughput sequencing and assembly

Paired-end and mate-pair sequencing was performed using the Illumina Sequencing Systems. Adaptor sequences, PCR duplicates, contaminants and low-quality sequences were removed, and high-quality sequences were assembled into contigs using ABySS (v1.3.5)^47^ and SOAP*de novo* (v1.05)^48^. Scaffolds were constructed from contigs using SSPACE (v-PREMIUM-2.3)^49^. Gaps in the scaffolds were closed using GapFiller (v1.10)^50^. Assembly completeness and redundancy were assessed with Core Eukaryotic Genes Mapping Approach^16^ and RNA-seq data (Supplementary Methods, section 2). Genome sizes were estimated by the k-mer-based method (Supplementary Fig. 18). For transcriptome analysis, high-quality RNA-seq reads were mapped to the genomes using TopHat (v2.0.12)^51^. Transcripts were built by Cufflinks (v2.0.2)^52^ with default settings. *De novo* assembly of RNA-seq reads was performed using Trinity (v2.0.3)^53^. Expression levels were evaluated by fragments per kilobase of transcript per million fragments of mapped genes, using Cufflinks (v2.0.2) (-G parameter) referenced to the final EVM integrated GFF files (Supplementary Methods, section 4).

### Gene prediction and annotation

We combined several approaches to predict protein-encoding genes, including homology-based searches, *ab initio* prediction and transcriptome-based prediction methods. Individual predictions were merged by EvidenceModeler (v1.1.1)^54^. Untranslated regions were added with PASA (v2.0.0)^55^. Predicted proteins were searched by BLAST algorithms for homologues in the National Center for Biotechnology Information non-redundant protein databases and Uniprot database. InterproScan5 (ref. 56) was used for identifying domains, mapping GO terms and assigning functional classifications. KO terms were assigned and pathway mapping was performed using the annotation tools at the Kyoto Encyclopedia of Genes and Genomes server (http://www.genome.jp/tools/kaas/) (see Supplementary Methods, section 5). Non-coding RNA and repeat elements were also predicted (Supplementary Methods, section 3.2).

### Comparative genomics and SNV analysis

Protein similarities were determined by all-against-all BLASTP searches (1*e*^−4^) using predicted proteome sequences of *T. asiatica* as queries against those of *T. saginata* and *T. solium*. The *T. solium* genome assembles (version 2) available at http://taenia.big.ac.cn/taenia/index.html were used in all analyses or otherwise as specified. The resulting *T. asiatica*-specific genes were further searched by BLASTP (1*e*^−3^, 80% length coverage) against genome assemblies of *T. saginata* and *T. solium* to identify high-confident-specific genes. The assemblies of the three human tapeworms were aligned using MUMmer (v3.22)^57^ to identify genome syntenic blocks. The collinearity analysis for orthologous genes on scaffolds was conducted using MCscanX^58^ (Supplementary Methods, section 6). High-quality reads were mapped to the reference assemblies using Bowtie2 (v2.2.3)^59^. Reads corresponding to PCR duplicates were removed by MarkDuplicates from PICARD (v1.119) (http://picard.sourceforge.net), followed by base quality recalibration and indel realignment by GATK (v3.5)^60^. SNVs and indels in the *T. asiatica* and *T. saginata* genomes were detected by HaplotypeCaller from GATK and filtered by the coverage, mapping quality score, FisherStrand (FS) value (Phred-scaled *P* value using Fisher's exact test) and other filters. To make the result comparable, a similar coverage (∼65 × ) of reads for each genome was used to calculate the proportion of variants removed by each filter (Supplementary Methods, section 3.3).

### Gene family construction and phylogeny analysis

Gene families were identified using protein sequences of *T. saginata*, *T. asiatica* and 8 other worms (that is., *T. solium*, *E. granulosus*, *E. multilocularis*, *Hymenolepis microstoma*, *Schistosoma japonicum*, *S. mansoni*, *Ascaris suum* and *C. elegans*; Supplementary Table 13) by OrthoMCL (v2.0.9)^61^ (Supplementary Figs 19 and 20). A maximum likelihood phylogenetic tree was inferred from a concatenated nucleotide data set containing 747 single-copy orthologous genes shared by all 10 species by RAxML (v8.0.24)^62^ with the best fit model (GTR+I+4-rate Γ for CDS; Supplementary Methods, section 7). The divergence dates were estimated with the relaxed-clock model using BEAST2 (v2.1.3)^23^ (Supplementary Methods, section 8).

### Gene family analysis

Gene family expansion and contraction were determined using the CAFÉ (v3.0)^63^ based on the phylogenetic tree constructed by RAxML (v8.0.24). The minimum ancestral gene families were determined using DOLLOP program included in the PHYLIP package (v3.695)^64^ to estimate gain/loss evolutions of gene families.

### Paralogous gene groups and GDs

An all-against-all protein sequence similarity search was performed using BLASTP (*e* value≤1*e*^−10^), followed by clustering the paralogous groups within each genome using Markov Clustering (MCL) (mclblastline pipeline)^65^. For each gene family, a protein alignment was constructed using MAFFT (v7.147b)^66^. This alignment was used as a guide for aligning the DNA sequences of gene family pairs, using ParaAT (v1.0)^67^. Paralogous gene pairs were retained if the two sequences were alignable over a length of >150 amino acids with an identity score of at least 30%. *K*_s_ value was calculated with the maximum likelihood estimation method using the program codeml (CodonFreq=2, runmodel=−2) in the PAML package (v4.8). Only *K*_s_ values ≤5 were retained for further analysis. An average linkage clustering approach was used to correct the redundancy of *K*_s_ values that correspond to GD events. The GD modes of each genome were estimated by MSCANX. Recently, duplicated genes (in-paralogs) along *T. asiatica* and *T. saginata* that arise after their divergence were determined by Inparanoid (v4.1)^68^. The Tajima's relative rate test for in-paralogous gene pairs was performed, using orthologous genes of *T. solium* as outgroup (supplementary Methods, section 8).

### Likelihood ratio tests for PSGs

A total of 6,581 one-to-one orthologous gene groups were extracted from genomes of the six tapeworms (*T. asiatica*, *T. saginata*, *T. solium*, *E. granulosus*, *E. multilocularis* and *H. microstoma*) to identify PSGs. Multiple protein-coding DNA sequence alignments were generated using ParaAT (v1.0)^67^ and MAFFT (v7.147b)^66^. All gaps in the alignments were removed, and likelihood ratio tests for selection (*P*<0.05) on each branch of the phylogenetic tree were performed using Codeml implemented in the PAML package (v4.8)^28^ with a modified branch-site model A (model=2, NSsites=2; Supplementary Methods, section 9).

### Identification of potential drug targets

Putative proteases and PIs were detected using the MEROPS batch BLAST server (http://merops.sanger.ac.uk/cgi-bin/batch_blast) (*E* value<1*e*^−4^). LGIC genes were identified by searching tapeworm homologues against the LGIC database (http://www.ebi.ac.uk/compneur-srv/LGICdb/) and annotated LGIC proteins from *E. multilocularis* and *S. mansoni*^13^,^15^. The resulting hits were used as BLASTP queries against the National Center for Biotechnology Information non-redundant database. Sequences homologous to LGIC-related proteins or having no hits in the non-redundant database were retained as putative LGICs. Protein kinase domain-containing proteins were extracted from InterProScan5 domain annotations. The corresponding domains were clustered with a reference domain data set (Human, fly and *C. elegans*; KINBASE; http://kinase.com/kinbase/FastaFiles/) using OrthoMCL. The non-assignable domains during the clustering were searched against other tapeworm kinases for classification. TM domains were first predicted by Phobius algorithm^69^ (length >250 aa). The resulting proteins with ≥3 and ≤15 TM domains were retained and searched by HMMER (v3.1b1)^70^ with HMMs against annotated GPCR sequences from Pfam database and from other tapeworms. Hits were further filtered by BLAST search against the non-redundant protein database (Supplementary Methods, section 11). Several bioinformatics tools were employed to identify classic and non-classic E/S proteins in the *T. asiatica* and *T. saginata* genomes (Supplementary Methods, section 10).

### Data availability

All sequence data that support the findings of this study have been deposited in GenBank with the following accession numbers: LWMK00000000 and LWMJ00000000 for Whole Genome Shotgun projects of *T. saginata* and *T. asiatica* under BioProject accession PRJNA71493 and PRJNA299871, respectively; SRR2890401, SRR2890402, SRR2890403, SRR2890404 and SRR2890405 for the *T. saginata* genome sequencing data; SRR2890205, SRR2890207, SRR2890209, SRR2890210, SRR2890211, SRR2890213, SRR2890214, SRR2890215 and SRR2890216 for the *T. asiatica* genome sequencing data; SRR2895139 and SRR2895068 for the transcriptome data of *T. saginata* and *T. asiatica*, respectively. The genome assemblies and annotations used in this study are also available at http://taenia.big.ac.cn/taenia/index.html. All other data supporting the findings of this study are available within the article and its Supplementary Information files, or from the corresponding authors on request.

## Additional information

**How to cite this article:** Wang, S. *et al*. Comparative genomics reveals adaptive evolution of Asian tapeworm in switching to a new intermediate host. *Nat. Commun.* 7:12845 doi: 10.1038/ncomms12845 (2016).

## Supplementary Material

Supplementary Information Supplementary Figures 1-20, Supplementary Tables 1-13, Supplementary Methods and Supplementary References.

Supplementary Data 1 GO enrichment analysis of the genes containing peak-2 introns in the *T. asiatica *and *T. saginata *genomes.

Supplementary Data 2 The in-paralog pairs showing differential evolution rates in the *T. asiatica *and *T. saginata *genomes.

Supplementary Data 3 GO enrichment analysis of heterozygous genes in the *T. asiatica *and *T. saginata *genomes.

Supplementary Data 4 Positively selected genes identified from *T. asiatica *and *T. saginata *lineage.

Supplementary Data 5 Proteases identified from the *T. asiatica *and *T. saginata *genomes.

Supplementary Data 6 Non-protease homologues in the *T. asiatica *and *T. saginata *genomes.

Supplementary Data 7 Protease inhibitors identified from the *T. saginata *and *T. asiatica *genomes.

Supplementary Data 8 GPCRs in the *T. asiatica *and *T. saginata *genomes.

Supplementary Data 9 Protein Kinases in the *T. asiatica *and *T. saginata *genomes.

Supplementary Data 10 Ligand-gated ion channels.

Supplementary Data 11 Potential drug targets specific to tapeworms compared with human.

Supplementary Data 12 Potential drug targets with homologs in drug target databases.

Supplementary Data 13 Potential nuclear molecular markers to differentiate *T. saginata *(TSA) and *T. asiatica *(TAS).

## Figures and Tables

**Table 1:** Genomic features of *T. saginata* and *T. asiatica* in comparison with other worms.

	***T. saginata***	***T. asiatica***	T. solium*	***E. multilocularis***	***H. microstoma***	***S. mansoni***	***C. elegans***
Assembly size (Mb)	169	168	131	114	141	365	100
GC content (%)	43.2	43.2	43.5	42.2	36.0	35.2	35.4
Coding genes number	13,161	13,323	11,902	10,506	10,141	10,772	20,469
Average gene length (Kb)	6.0	5.9	4.6	5.4	6.1	15.4	3.1
Protein length (aa)	464	466	444	505	490	477	453
Gene density (genes per Mb)	77.9	79.3	90.9	92.2	71.9	29.5	201.0
Number of exons per gene	6.2	6.2	6.6	6.8	6.4	6.5	6.4
Mean length of exons (bp)	237	244	237	220	229	226	212
Number of introns per gene	5.2	5.2	5.6	5.8	5.4	5.5	5.4
Mean length of introns (bp)	864	831	775	684	862	2,460	354
GC content of exon (%)	49.7	49.6	50.2	50.0	44.3	36.0	42.6
GC content of intron (%)	41.5	41.2	40.8	39.9	34.6	34.7	32.5
Repeat content (%)	10.4	10.9	18.1	10.9	7.6	40.0	17.0
tRNA number	339	353	162	856	44	153	966

^*^T. solium v2 genome (China isolate) properties and gene models.
