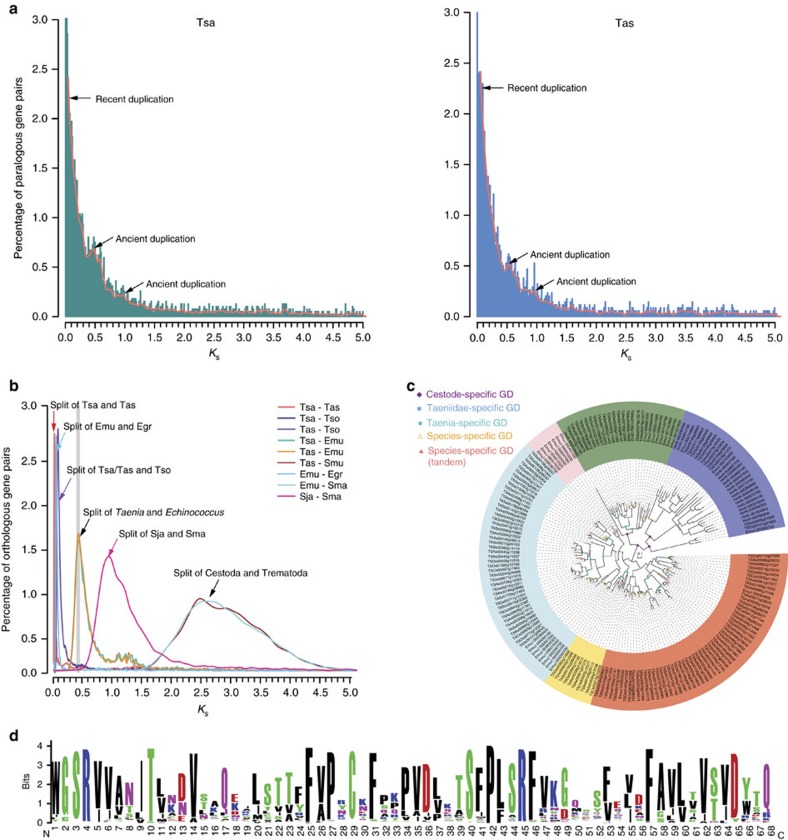# Erratum: Comparative genomics reveals adaptive evolution of Asian tapeworm in switching to a new intermediate host

**DOI:** 10.1038/ncomms13469

**Published:** 2016-11-03

**Authors:** Shuai Wang, Sen Wang, Yingfeng Luo, Lihua Xiao, Xuenong Luo, Shenghan Gao, Yongxi Dou, Huangkai Zhang, Aijiang Guo, Qingshu Meng, Junling Hou, Bing Zhang, Shaohua Zhang, Meng Yang, Xuelian Meng, Hailiang Mei, Hui Li, Zilong He, Xueliang Zhu, Xinyu Tan, Xing-quan Zhu, Jun Yu, Jianping Cai, Guan Zhu, Songnian Hu, Xuepeng Cai

Nature Communications **7:**: Article number: 12485; DOI: 10.1038/ncomms12845 (2016); Published: 09 22 2016; Updated: 11 03 2016

This Article contains errors in Fig. 3. In panel b, the axes are displaced relative to the data, and in panel c, the label ‘Species-specific GD' in red should read ‘Species-specific GD (tandem)'. The correct version of Fig. 3 appears below as Fig. 1.

## Figures and Tables

**Figure 1:**