# Supplementary material for: Comparative genomics reveals adaptive evolution of Asian tapeworm in switching to a new intermediate host
Source: Nat Commun. 2016 Sep 22;7:12845. doi: 10.1038/ncomms12845 (PMC5036155; doi:10.1038/ncomms12845)
Supplement: Supplementary Information — Supplementary Figures 1-20, Supplementary Tables 1-13, Supplementary Methods and Supplementary References. [file ncomms12845-s1.pdf]

Supplementary Figures

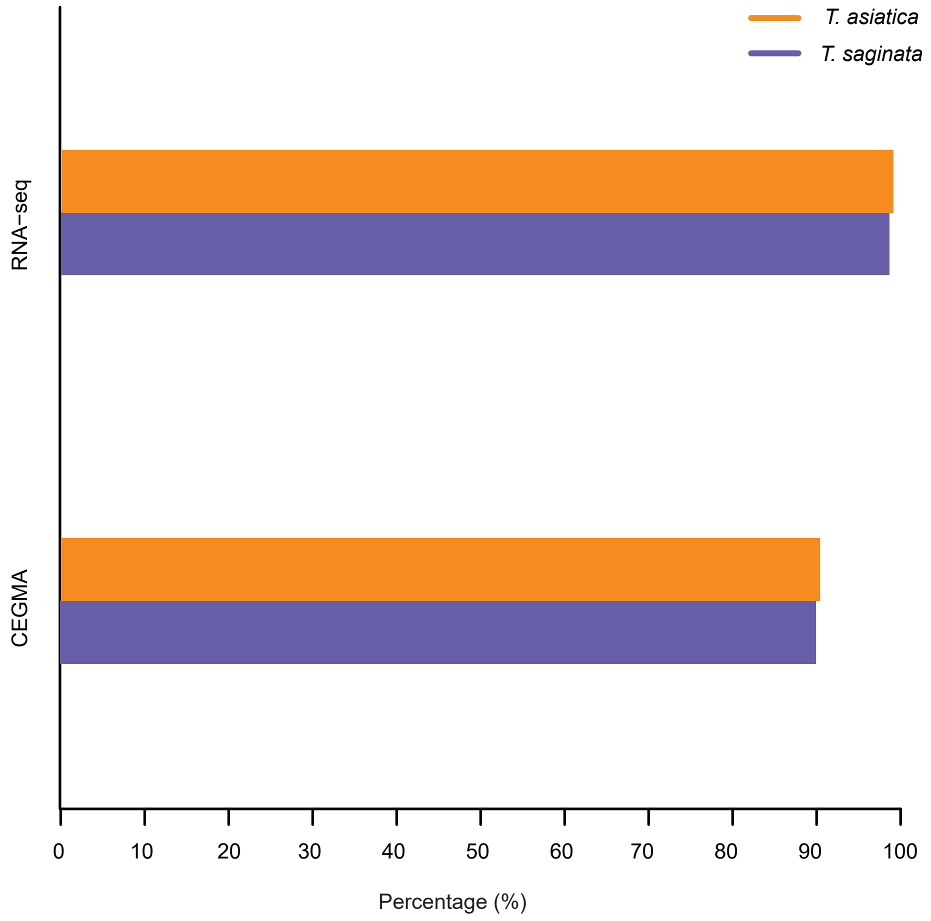

Supplementary Figure 1. Validation of the *T. saginata* and *T. asiatica* genome assemblies. cDNAs constructed from RNA-Seq data by Trinity were used to evaluate the gene-coding regions of the assemblies, in which more than 98% of cDNA sequences were covered by the assemblies. The completeness of the assemblies was also evaluated using the CEGMA (Core Eukaryotic Genes Mapping Approach) pipeline, in which 89.52% (*T. saginata*) and 90.32% (*T. asiatica*) of the 248 core eukaryotic genes were mappable in the two draft genomes.

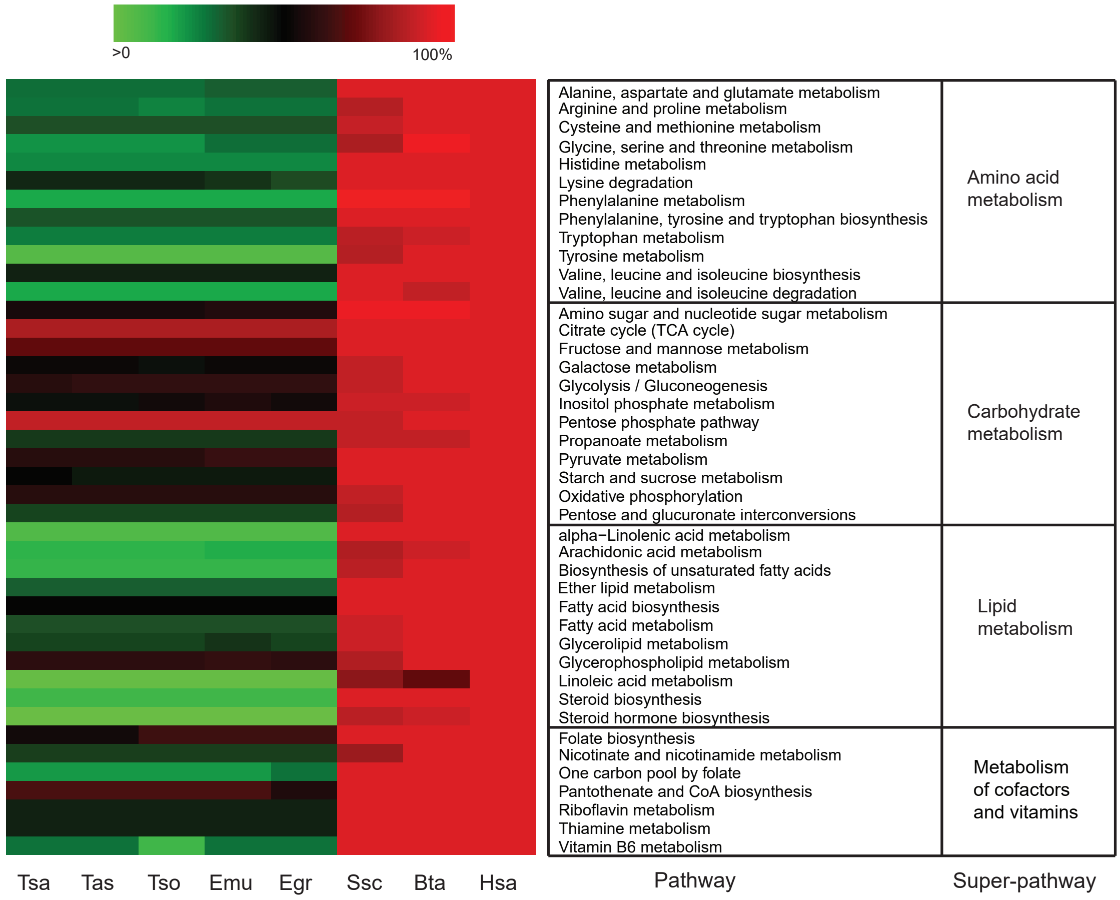

Supplementary Figure 2. Metabolic pathways in *T. saginata* and *T. asiatica* compared with those in other tapeworms and their hosts. The heatmap was drawn based on KEGG (Kyoto Encyclopedia of Genes and Genomes) term assignments using BLASTP method. Each row indicates an individual metabolic pathway. Super-pathways are grouped by their super-class membership defined by KEGG. Colored tiles indicate the ratio of components within each species in comparison with those within human for each pathway. The individual metabolic pathways among *T. saginata* (Tsa), *T. asiatica* (Tas), *T. solium* (Tso), *E. multilocularis* (Emu), and *E. granulosus* (Egr) are highly conserved and the abilities of synthesis in these tapeworms are reduced in comparison with those in *Bos taurus* (Bta), *Sus scrofa* (Ssc) and *Homo sapiens* (Hsa).

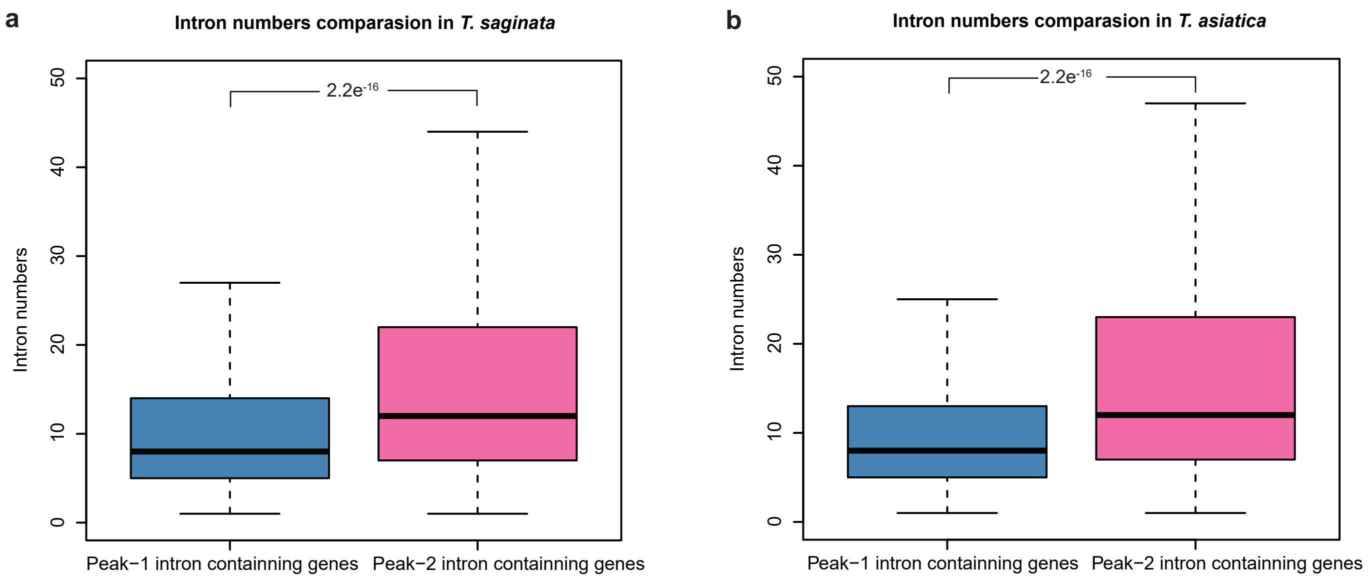

Supplementary Figure 3. Intron numbers of peak-1/2 intron containing genes in *T. saginata* and *T. asiatica*. Genes containing peak-2 introns (15.05 and 14.80 introns per gene in *T. asiatica* and *T. saginata*, respectively) tend to possess more introns than those containing peak-1 introns (10.15 and 10.52) (p-value = $2.2e^{-16}$ by two-sided Wilcoxon rank sum test). The whiskers are lines that extend from box to highest and lowest values, excluding outliers determined by default R code.

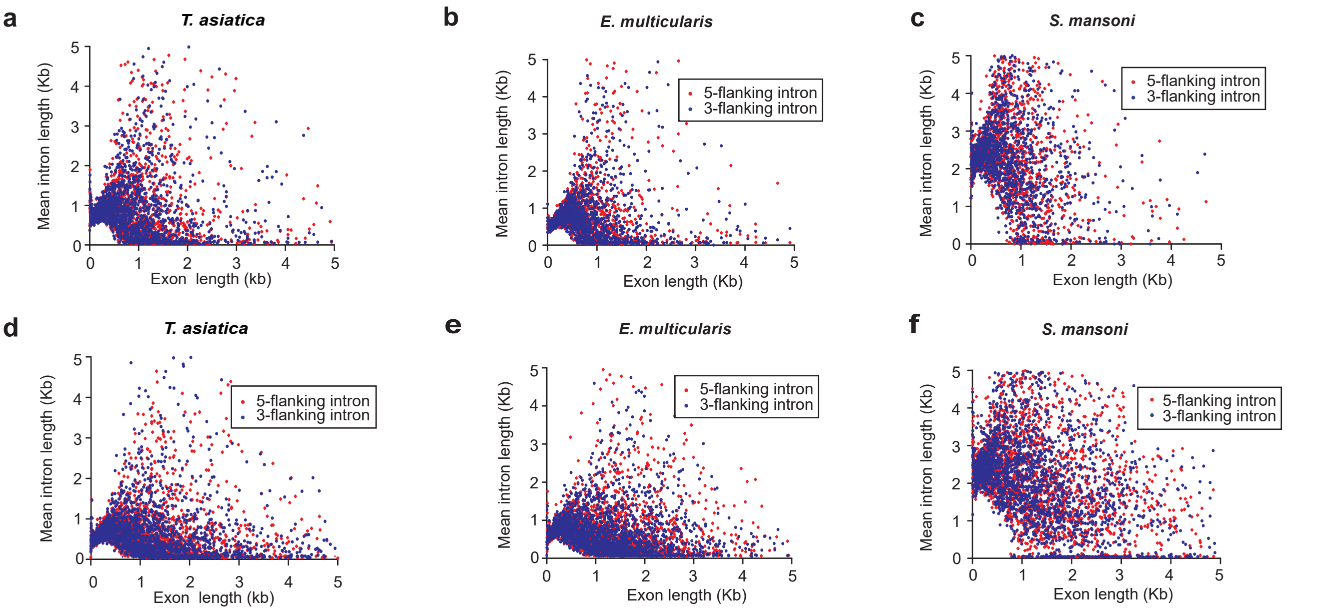

Supplementary Figure 4. Mean length preference of introns flanking small exons in the worms. The predicted gene models (a-c) and transcripts assembled by RNA-seq data (d-f) were used in this study. The minimal mean lengths of intron pairs flanking a small exon length (<400 bp) are 370 bp (*T. asiatica*) long. The mechanism behind this phenomenon still remains to be illustrated.

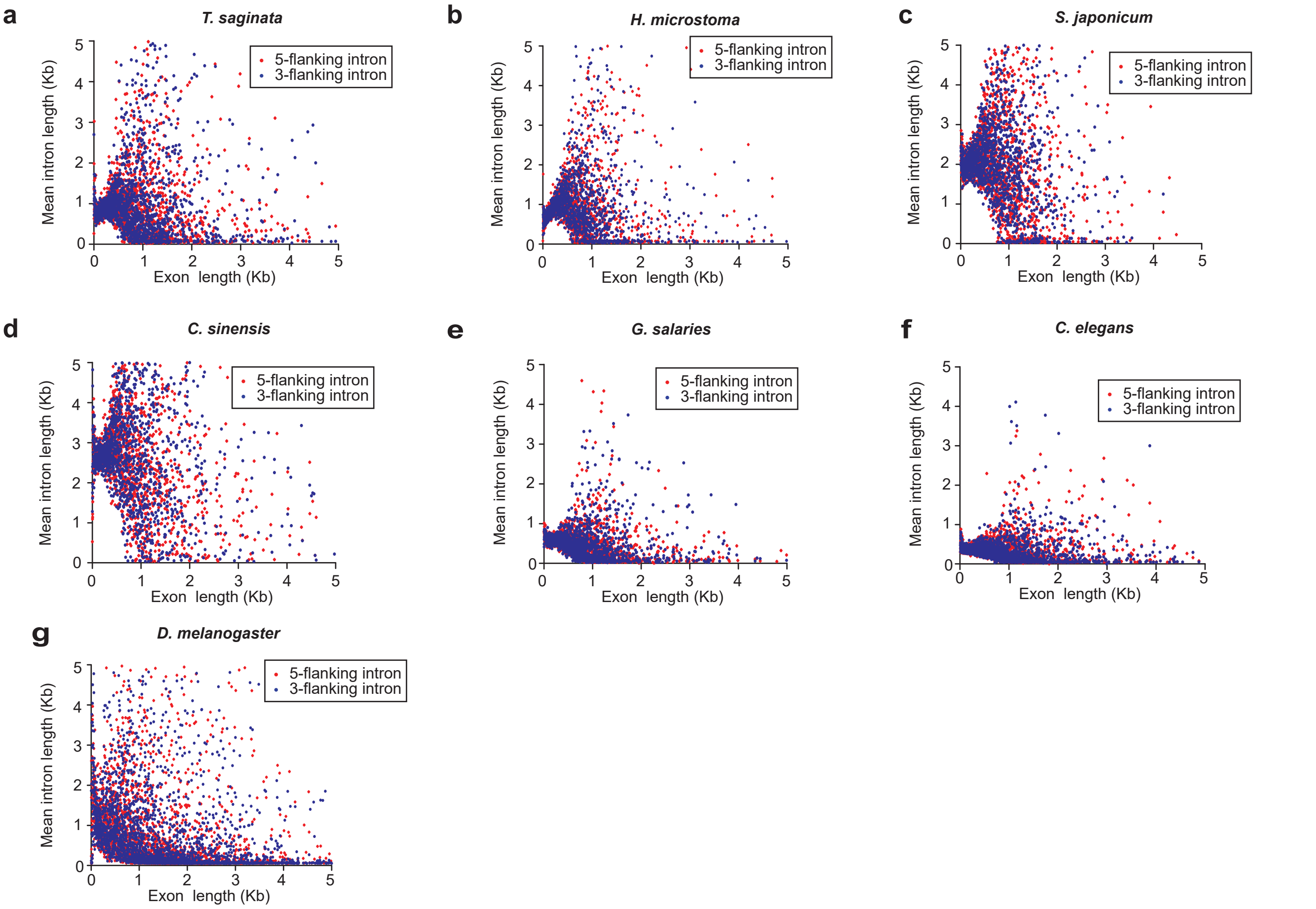

Supplementary Figure 5. Mean length preference of introns flanking small exons in the parasitic worms and outgroups. The predicted gene models (a-e) and the manually refined gene models (f and g) were used in the analysis. The minimal mean lengths of introns flanking small exons appear to vary in different species.

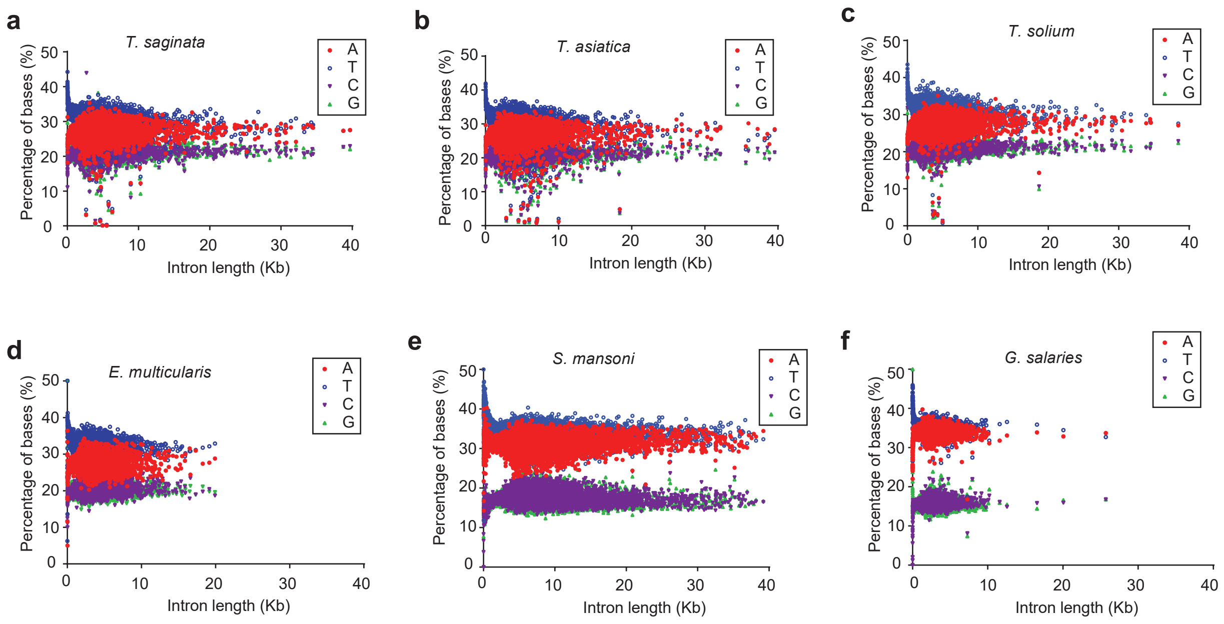

Supplementary Figure 6. A/T usage bias in nucleotide compositions of introns in the parasitic flatworms. Introns in tapeworms are A/T bases-rich (e.g., 26.8%/31.1% in *T. saginata*; 26.6%/31.1% in *T. asiatica*), but not as disproportional as in *S. mansoni* (31.3%/33.6%) and *G. salaris* (33.8%/35.3%).

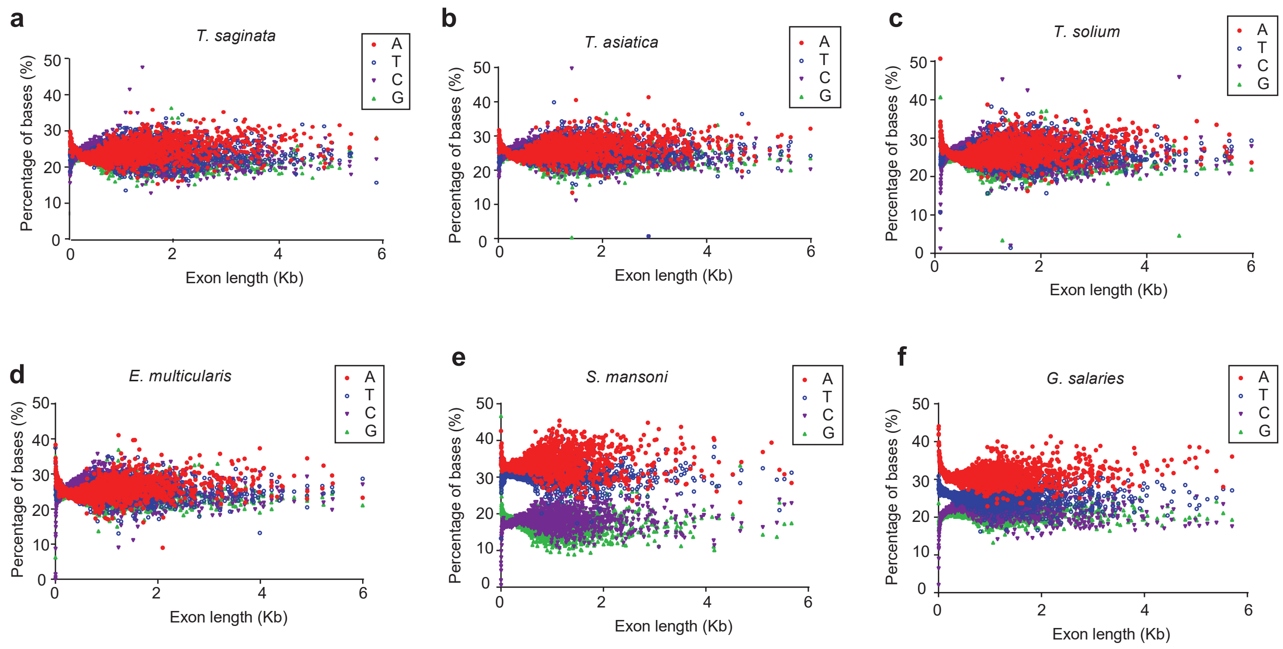

Supplementary Figure 7. A/T usage bias in nucleotide compositions of exons in the parasitic flatworms. No significant A/T-bias in exons was observed in tapeworms, although it was found in the flukes (i.e., 25.2%/25.0% in *T. saginata* and 25.3%/25.1% in *T. asiatica*, vs. 31.5%/33.2% in *S. mansoni*).

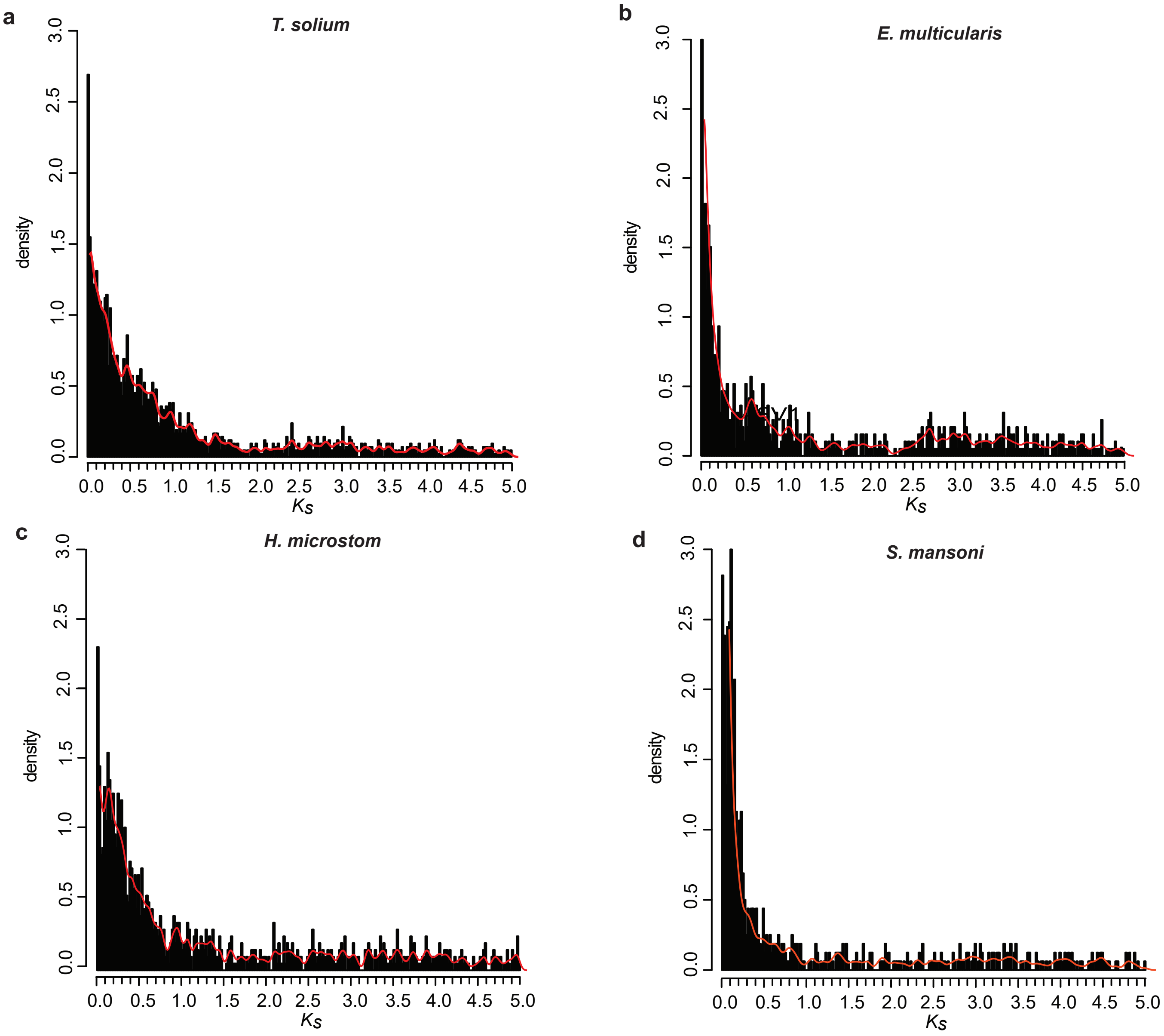

Supplementary Figure 8. *Ks* distribution of paralogous genes in parasitic flat worms. Distribution of *Ks* values of paralogous genes in the *T. solium* (a), *E. multicularis* (b), *H. microstom* (c) and *S. mansoni* (d) genomes.

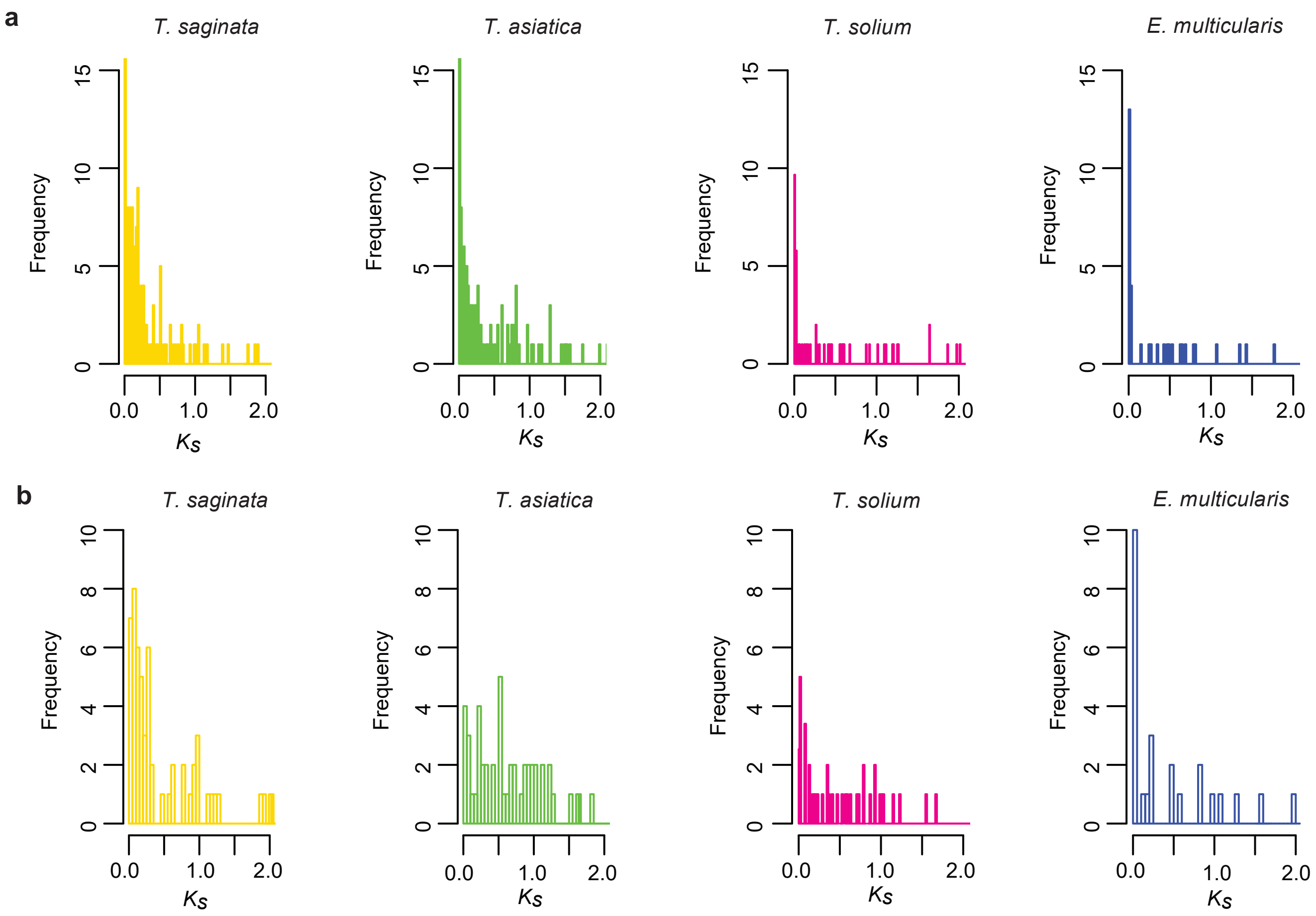

Supplementary Figure 9. Extensive duplications of large paralogous gene groups. (a) *Diagnostic antigen gp50* gene family and (b) *HSP70* gene family experienced continuous and extensive gene duplication during the evolution history of the tapeworm lineage.

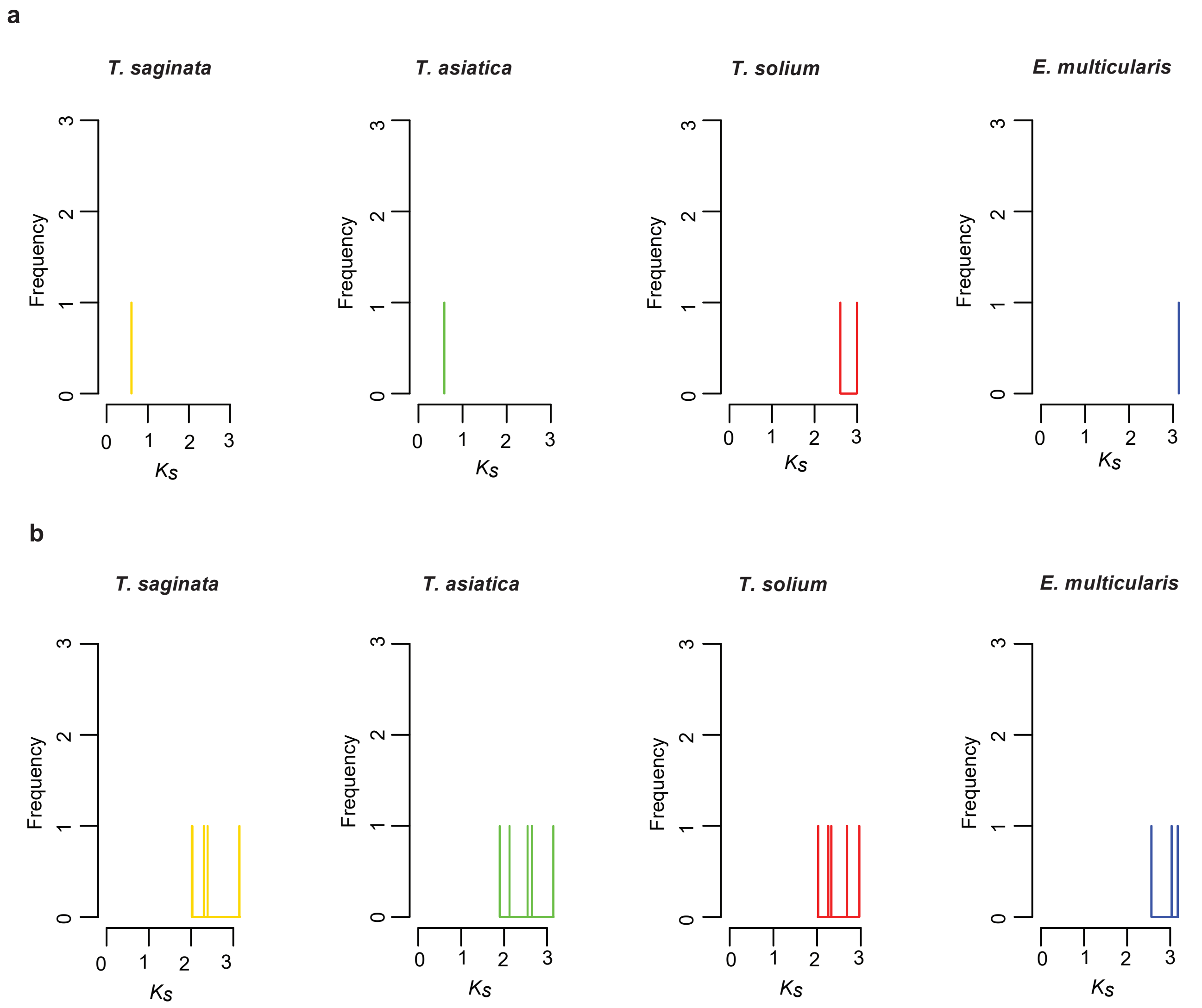

Supplementary Figure 10. Duplications of house-keeping genes. (a) ATP dependent RNA helicase gene family and (b) *Tubulin* gene family have experienced rare gene duplications during the evolution history of the tapeworm lineage.

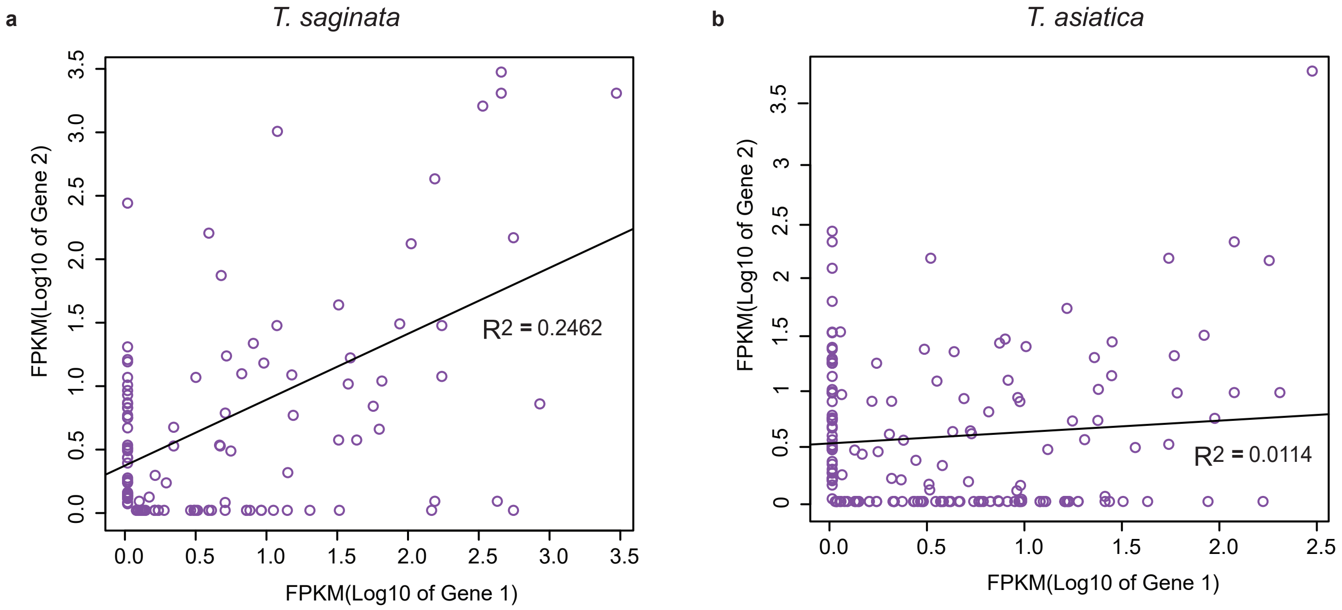

Supplementary Figure 11. Expression patterns of in-paralogous gene pairs. Pearson correlation coefficient between expression levels (FPKM value) of an in-paralogous gene pair was calculated.

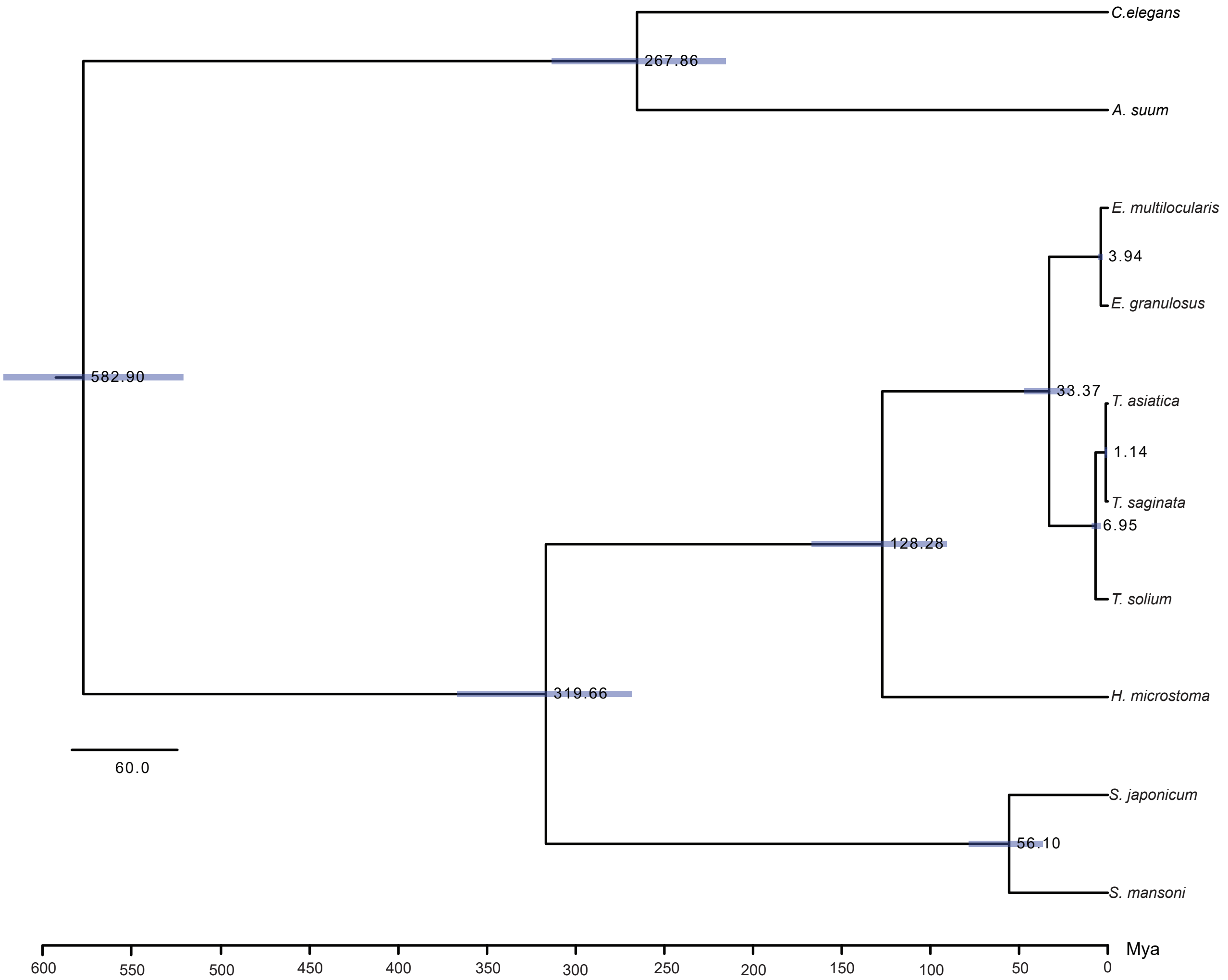

Supplementary Figure 12. Divergence time of the tapeworms and related worms. The evolutionary history of worms was reconstructed and the timings were estimated by BEAST2 using relaxed log normal model. The number at the node represents the divergence time between two lineages. The blue bar represents interval of 95% highest probability density.

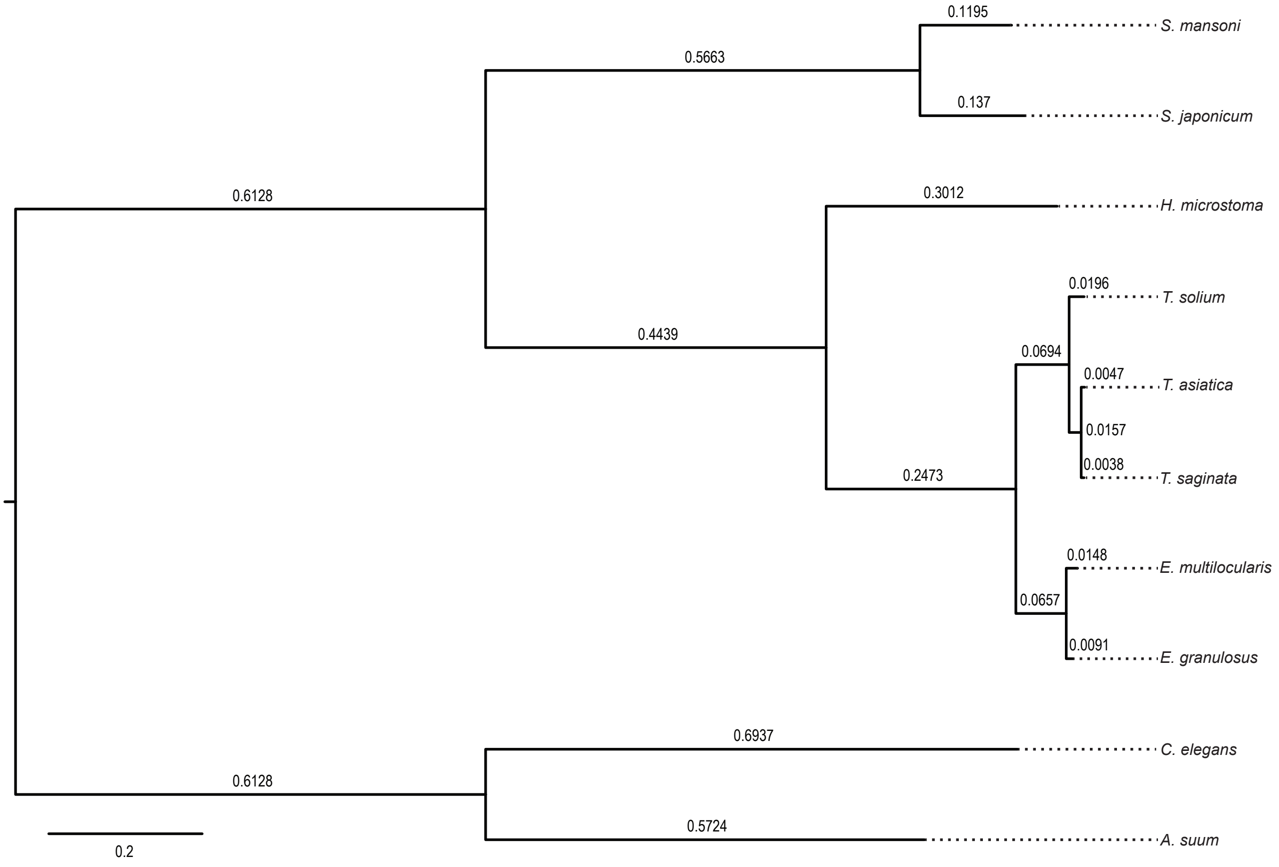

Supplementary Figure 13. Phylogenetic relationships of tapeworms and related species. The phylogeny of the 10 helminthes was reconstructed based on the CDS sequences of the 747 single-copy genes shared among all the worms with *C. elegans* and *A. suum* as outgroups, by RAxML (settings: GTR+I+G). The branch lengths (solid line) are indicated by the values above the branches.

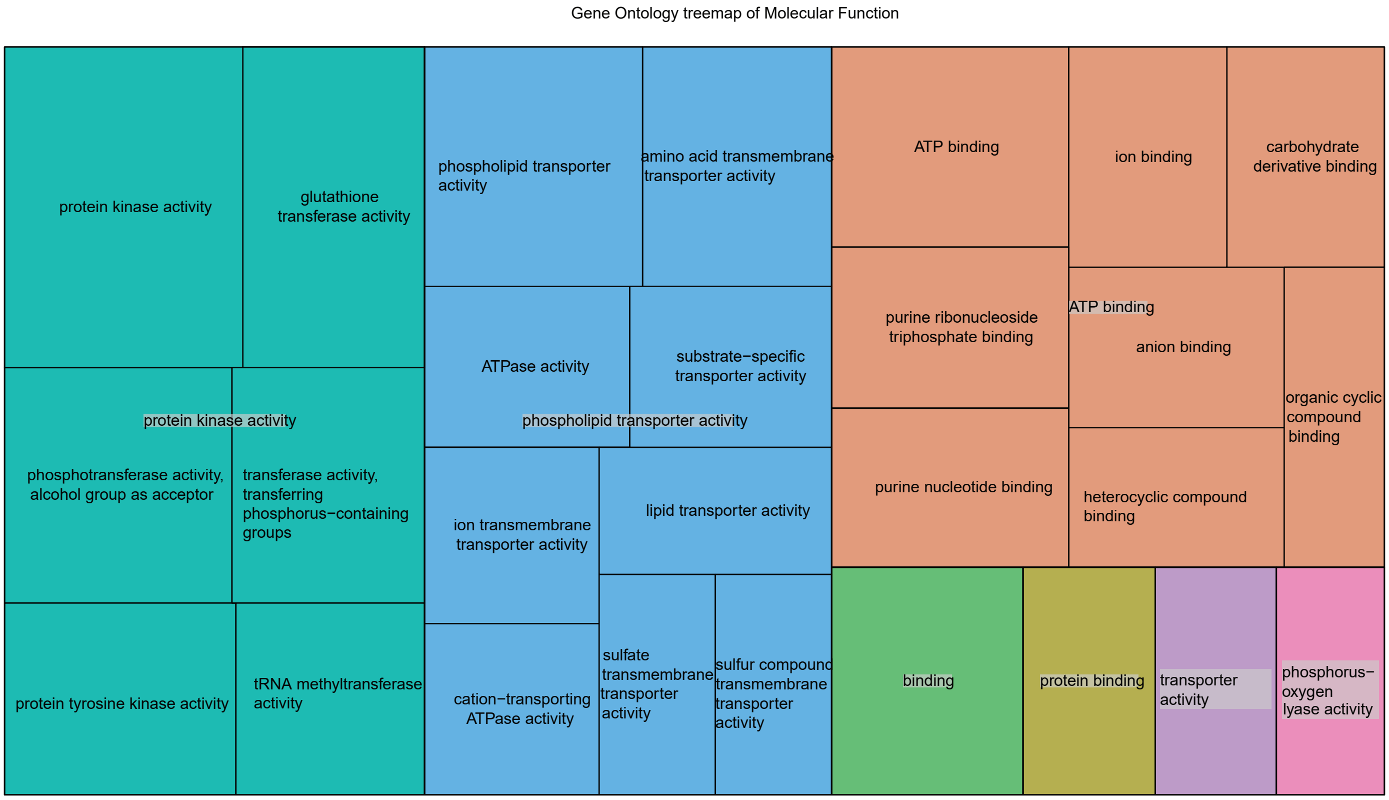

Supplementary Figure 14. GO enrichment analysis of genes with SNVs in the *T. asiatica* genome. In the *T. asiatica* genome, 1,170 genes were detected to have high quality SNVs. The genes that have classifications in the Gene Ontology database are used for GO enrichment analysis with BLAST2GO and the results were further summarized by REVIGO. Each rectangle is a single cluster representative, and its function is shown. The representatives are joined into 'superclusters' of loosely related terms and visualized with different colors. Sizes of the rectangles were drawn to reflect the p-values in enrichment analysis.

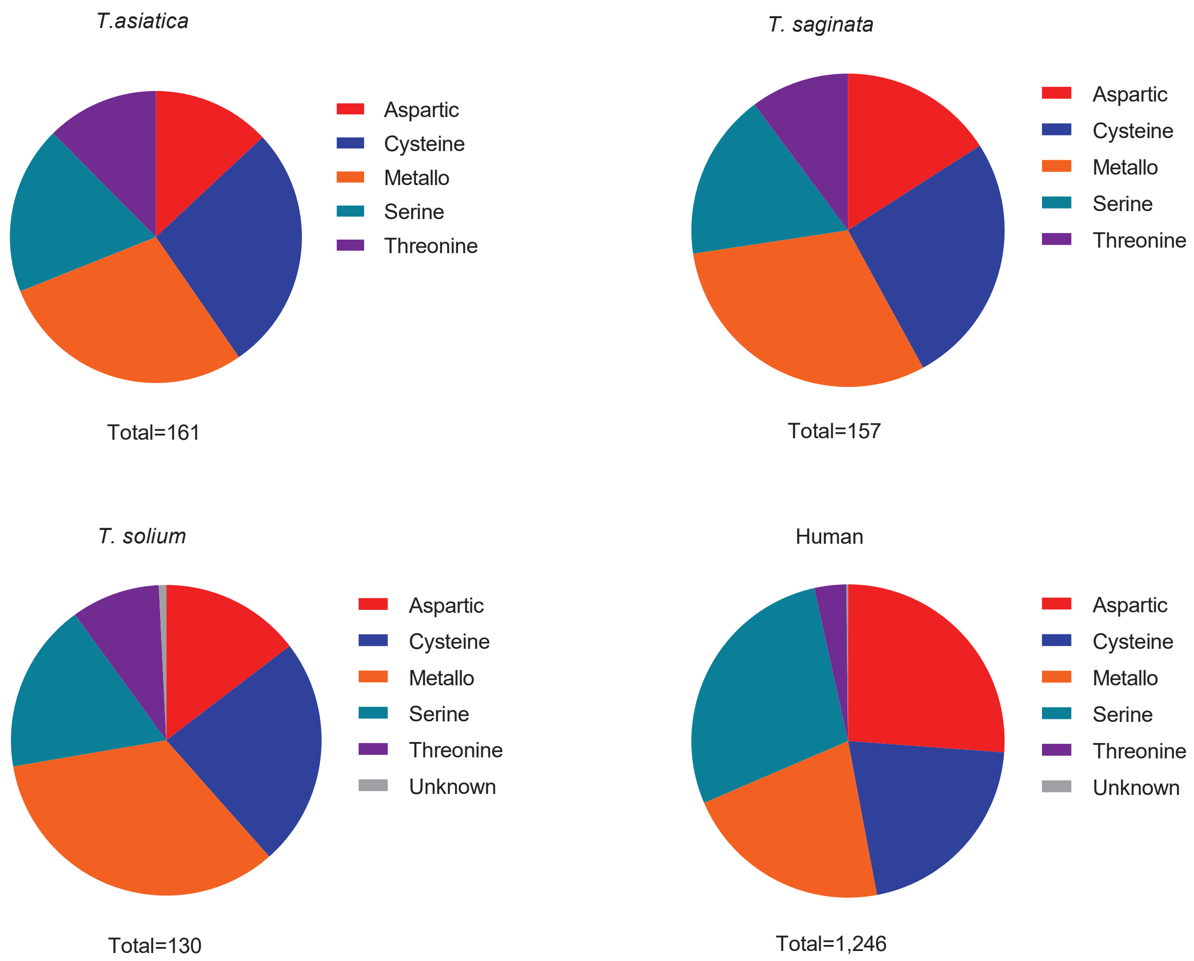

Supplementary Figure 15. Comparison of proteases in the human tapeworms and human genomes. The proteases of human were retrieved from the MROPS database (http://merops.sanger.ac.uk/).

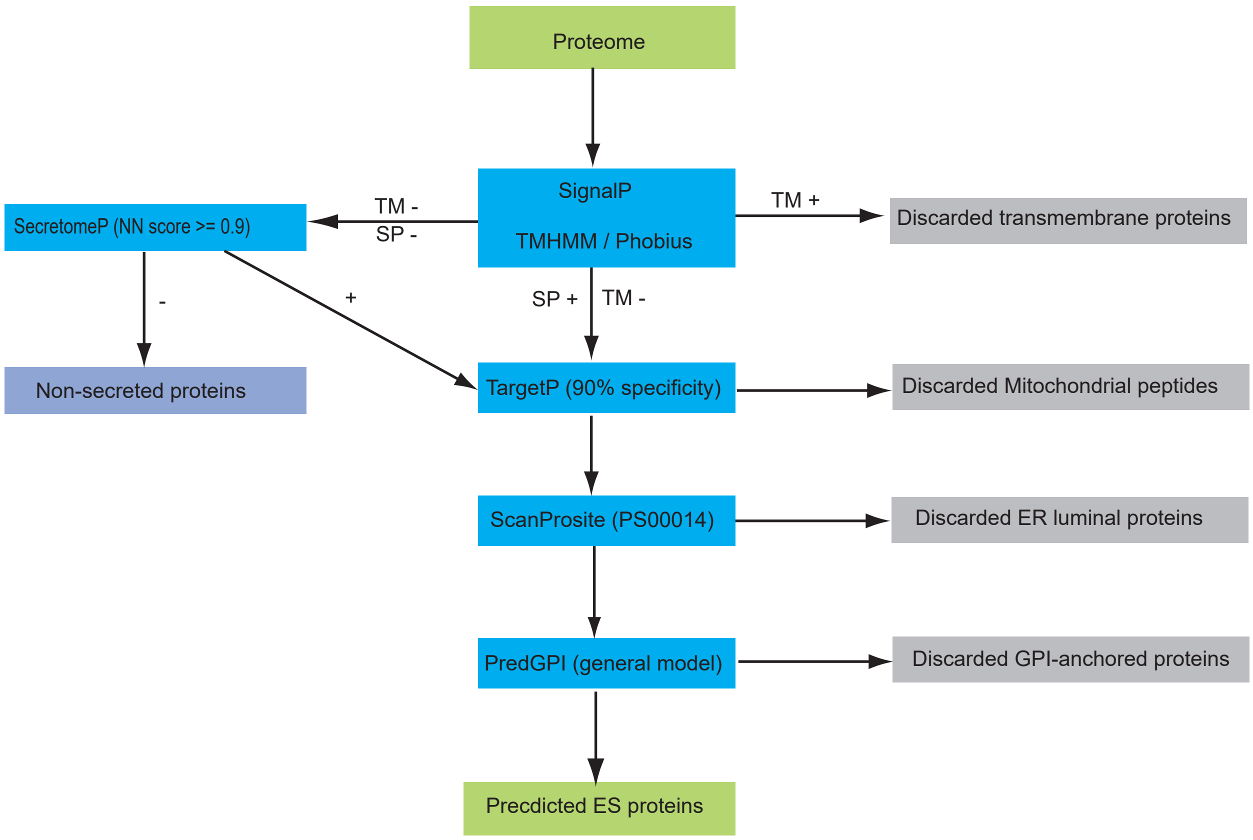

Supplementary Figure 16. The workflow for E/S protein identification in the *T. asiatica* and *T. saginata* genomes. The integration of bioinformatics tools were used to refine the E/S protein prediction, by predicting the classical and non-classical secreted proteins and excluding the proteins with transmembrane regions, ER luminal signals, mitochondrial location signals and GPI-anchored signals.

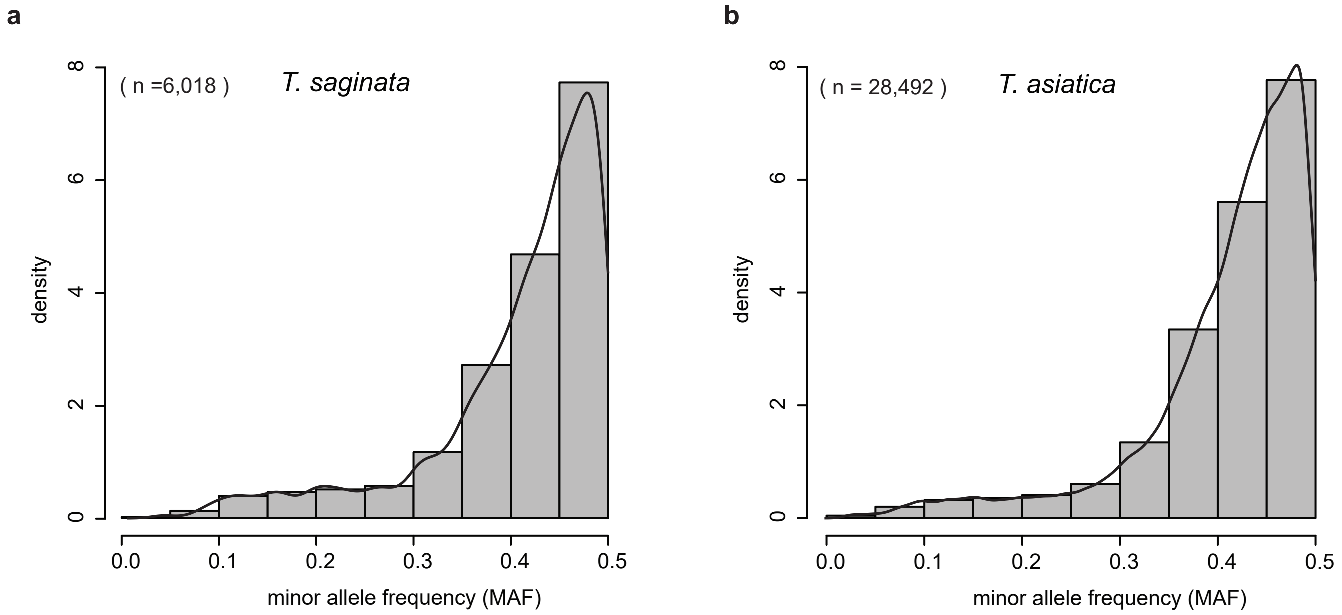

Supplementary Figure 17. Distribution of minor allele frequency of *T. saginata* and *T. asiatica*. The distribution of MAF peaks at 0.5 for each genome, which indicated that it is diploid.

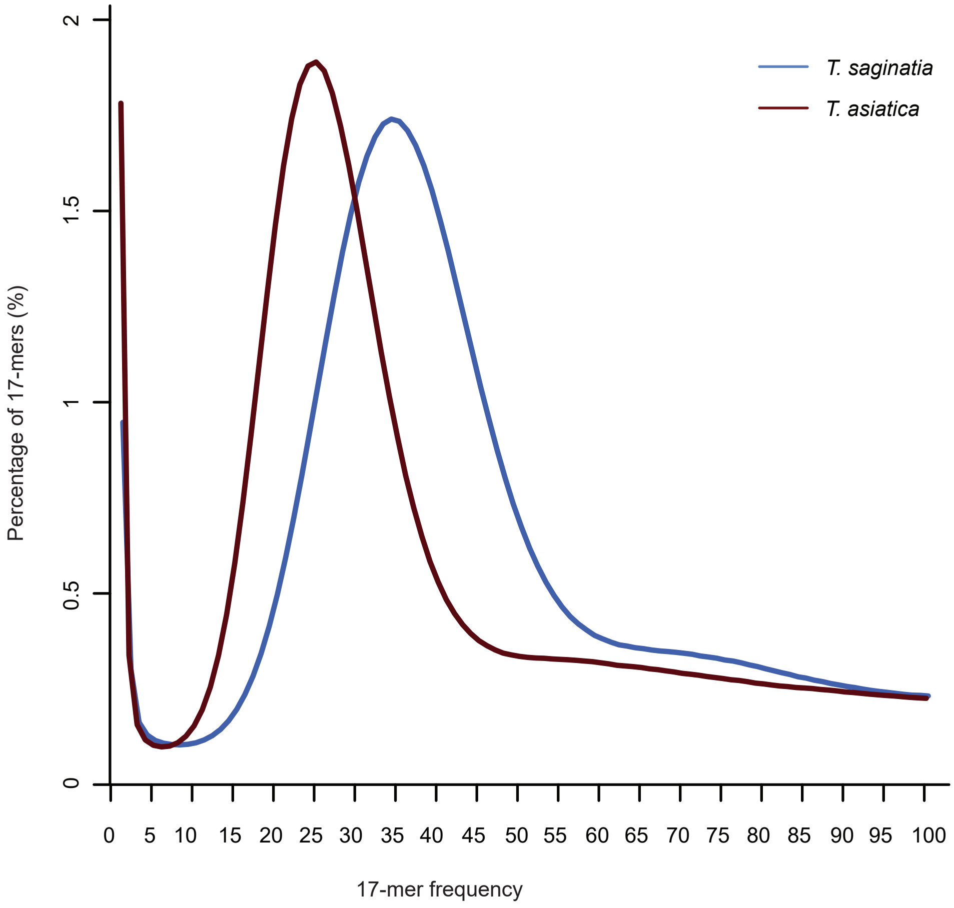

Supplementary Figure 18. Frequency distribution of 17-mers of the sequenced pair-end reads. The peak depth of error-free 17-mers in *T. saginata* is at ~34×, and that in *T. asiatica* is at ~25×. The genome sizes of the tapeworms were estimated with the formula: M=N*(L-K+1)/L and G=T/N, where T represents the total high-quality sequence bases, N represents the actual sequencing depth, L represents the average length of reads, K represents the k-mer length, M represents the peak in the distribution of the 17-mer frequencies, and G represents the estimated genome size.

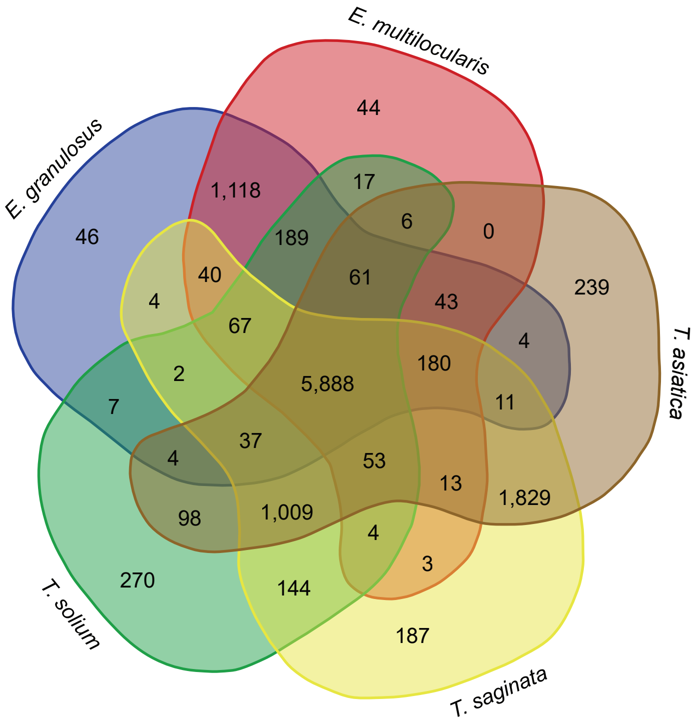

Supplementary Figure 19. Venn diagram of gene families in *T. saginata* and *T. asiatica* in comparison with those in other tapeworms. The gene families were determined by OrthoMCL.

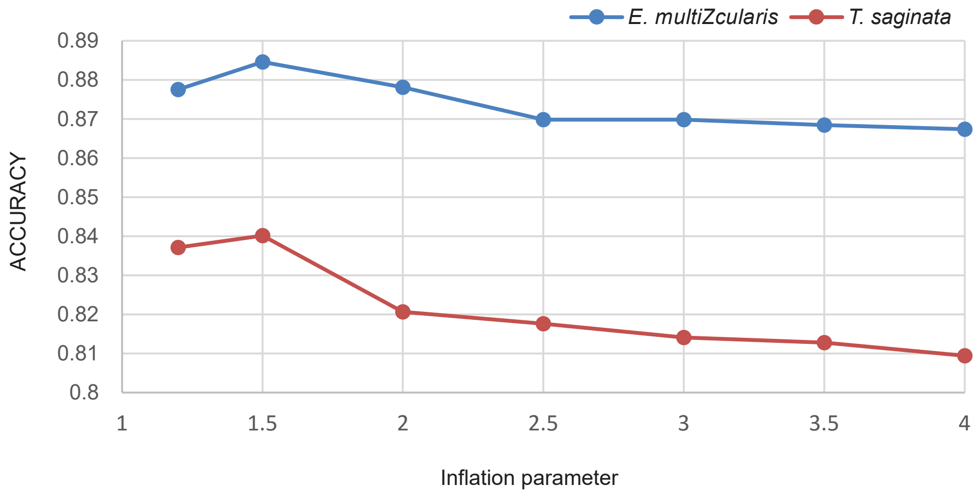

Supplementary Figure 20. The ACCURACY curve of EC-annotated sequences for different inflation parameter values in OrthoMCL algorithm. The ACCURACY [(TP + TN)/(TP + TN + FP + FN)] of the OrthoMCL algorithm (shown on the Y-axis) is plotted against the range of the inflation parameter values (shown on the X-axis).

Supplementary Tables

Supplementary Table 1. Statistics of the *T. saginata* and *T. asiatica* assemblies.

	T. saginatia		***T. asiatica***	
	**Contig**	**Scaffold**	**Contig**	**Scaffold**
Sequence Count	6,865	3,626	10,754	6,904
Maximum Size (bp)	1,502,447	7,334,011	1,097,630	4,220,646
Minimum Size (bp)	100	500	100	500
Mean Size (bp)	24,228	46,636	15,480	24,432
N50 Size (bp)	137,526	586,235	110,486	342,420
N50 Number	276	66	319	102
N90 Size (bp)	14,249	29,426	8,453	14,318
N90 Number	1,800	751	2,596	1,589
Total gap (bp)	-	2,788,521	-	4,273,122
Average GC(%)	43.19%	43.19%	43.15%	43.15%
Total Length(bp)	166,327,247	169,104,303	164,405,424	168,683,493

Supplementary Table 2. Summary of repeat elements identified in the *T. saginata* and *T. asiatica* genomes.

	T. saginata			***T. asiatica***		
**Class**	**Number**	**Length**	**Percentage**	**Number**	**Length**	**Percentage**
LTR	2,071	3,414,414	2.02%	1,390	2,533,366	1.50%
LINE	84	151,847	0.09%	87	289,178	0.17%
SINE	212	253,336	0.15%	300	441,445	0.26%
DNA element	69	116,145	0.07%	190	353,413	0.21%
Tandem Repeat	27,668	1,478,078	0.87%	23,084	1,051,902	0.62%
Unclassified	24,909	12,133,672	7.18%	23,594	12,619,692	8.13%
Total repeat	55,013	17,547,492	10.38%	48,645	17,288,996	10.90%
Assembled genome	-	169,104,303	100.00%	-	168,683,493	100.00%

Supplementary Table 3. Non-coding RNAs in the *T. asiatica* and *T. saginata* genomes.

Kind	Rfam ID	*T. saginata*	*T. asiatica*
**Ribosomal RNAs**			
5_8S_rRNA	RF00002	2	3
5S_rRNA	RF00001	11	10
SSU_rRNA_eukarya	RF01960	3	3
18S_rRNA		1	1
28S_RNA		1	1
**tRNAs**			
tRNA	RF00005	339	353
tRNA-Sec	RF01852	37	25
**Small nucleolar RNA (snRNA)**			
SNORA13	RF00396	0	1
snoR125	RF02409	1	
SNORA38	RF00428	1	1
SNORA79	RF00600	1	1
SNORA9	RF00411	1	1
SNORD31	RF00089	1	1
snoZ7	RF00268	0	1
snR86	RF01272	1	2
**spliceosomal RNA**			
U11	RF00548	1	1
U12	RF00007	1	1
U1	RF00003	36	35
U2	RF00004	21	37
U3	RF00012	0	1
U4atac	RF00618	1	1
U4	RF00015	3	1
U5	RF00020	2	1
U6	RF00026	5	4
**RNA component of signal-recognition particle (SRP)**			
Metazoa_SRP	RF00017	7	8
**nuclear ribonuclease P (RNase P)**			
RNaseP_nuc	RF00009	1	1
**MicroRNAs**			
miRNA		61	69

Supplementary Table 4. Gene duplication modes in the tapeworm genomes.

	T. saginata	***T. asiatica***	***T. solium***	***E. multilocularis***
Dispersed	5,195	5,543	4,036	2,987
Proximal	776	665	553	482
Tandem	1,205	1,173	771	644
Segmental	44	95	8	158
Total	7,220	7,476	5,368	4,271

The modes of gene duplications were consistent with the definations in MCSCANX.

Supplementary Table 5. Paralogous gene groups with continuous duplications in the *T. asiatica* genome.

Homologous Group	Duplication frequency	Function*
Group1	84	RNA directed DNA polymerase (reverse transcriptase)
Group2	68	heat shock protein 70
Group3	43	zinc finger protein
Group4	34	cyclin dependent kinase
Group5	52	diagnostic antigen gp50
Group7	39	expressed conserved protein
Group8	38	ryanodine receptor 44f
Group10	31	agc family protein kinase
Group12	27	UBiquitin Conjugating enzyme family member
Group14	24	retinal guanylyl cyclase 2
Group15	23	puromycin sensitive aminopeptidase
Group16	25	hypothetical protein
Group17	27	urea active transporter protein
Group18	26	hypothetical protein
Group25	24	T box transcription factor TBX6
Group30	25	Fibronectin, type III
Group31	19	homeobox protein Nkx 2.8/2.2
Group32	19	histone H2B
Group36	16	S phase kinase associated protein 1
Group39	14	aldo keto reductase family 1, member B4
Group40	15	beta 13 n galactosyltransferase
Group41	15	glioma pathogenesis protein 1
Group46	18	aminoacyl tRNA synthase complex interacting
Group55	14	Phosphotidylinositol phosphatase PTPRQ
Group64	14	EG95

* The function for each homologous group is shown with the majority of the annotations of group members. Only gene duplication events with ds value <2.6 were counted.

Supplementary Table 6. Comparison of enriched GO categories of in-paralogs in the *T. asiatica* and *T. saginata* genomes. Only GO terms with false discovery rate (FDR) < 0.05 in the two-sided Fisher's Exact Test are shown.

GO-ID	Term	Category	FDR (in *T. saginata*)	FDR (in *T. asiatica*)
GO:0000785	chromatin	C	-	1.03E-03
GO:0000786	nucleosome	C	-	1.03E-03
GO:0032993	protein-DNA complex	C	-	1.03E-03
GO:0044815	DNA packaging complex	C	-	1.03E-03
GO:0044427	chromosomal part	C	-	5.13E-03
GO:0005694	chromosome	C	-	1.14E-02
GO:0005858	axonemal dynein complex	C	-	1.32E-02
GO:0005930	axoneme	C	-	1.32E-02
GO:0044441	ciliary part	C	-	1.32E-02
GO:0044447	axoneme part	C	-	1.32E-02
GO:0044463	cell projection part	C	-	1.32E-02
GO:0097014	ciliary plasm	C	-	1.32E-02
GO:0005929	cilium	C	-	3.59E-02
GO:0046982	protein heterodimerization activity	F	-	1.03E-03
GO:0046983	protein dimerization activity	F	-	3.38E-03
GO:0004402	histone acetyltransferase activity	F	-	1.43E-02
GO:0034212	peptide N-acetyltransferase activity	F	-	1.55E-02
GO:0061733	peptide-lysine-N-acetyltransferase activity	F	-	1.55E-02
GO:0003777	microtubule motor activity	F	-	1.95E-02
GO:0008378	galactosyltransferase activity	F	1.16E-04	-
GO:0006334	nucleosome assembly	P	-	1.03E-03
GO:0031497	chromatin assembly	P	-	1.03E-03
GO:0034728	nucleosome organization	P	-	1.03E-03
GO:0065004	protein-DNA complex assembly	P	-	1.03E-03
GO:0071824	protein-DNA complex subunit organization	P	-	1.03E-03
GO:0006333	chromatin assembly or disassembly	P	-	1.10E-03
GO:0006323	DNA packaging	P	-	1.59E-03
GO:0071103	DNA conformation change	P	-	3.38E-03
GO:0006325	chromatin organization	P	-	6.20E-03
GO:0003341	cilium movement	P	-	6.50E-03
GO:0000054	ribosomal subunit export from nucleus	P	-	1.43E-02
GO:0000055	ribosomal large subunit export from nucleus	P	-	1.43E-02
GO:0033750	ribosome localization	P	-	1.43E-02
GO:0033753	establishment of ribosome localization	P	-	1.43E-02
GO:0042273	ribosomal large subunit biogenesis	P	-	1.43E-02
GO:0071428	rRNA-containing ribonucleoprotein complex	P	-	1.43E-02
GO:0007018	microtubule-based movement	P	-	2.17E-02
GO:0051276	chromosome organization	P	-	2.26E-02

GO:0006928	movement of cell or subcellular component	P	-	2.70E-02
GO:0051640	organelle localization	P	-	4.14E-02
GO:0051656	establishment of organelle localization	P	-	4.14E-02
GO:1901135	carbohydrate derivative metabolic process	P	2.03E-02	-
GO:0006486	protein glycosylation	P	1.16E-03	-
GO:0009100	glycoprotein metabolic process	P	1.16E-03	-
GO:0009101	glycoprotein biosynthetic process	P	1.16E-03	-
GO:0043413	macromolecule glycosylation	P	1.16E-03	-
GO:0044723	single-organism carbohydrate metabolic process	P	6.80E-03	-
GO:0070085	glycosylation	P	1.16E-03	-

Supplementary Table 7. Heterozygous single nucleotide variations (SNVs) and small indels in the *T. saginata* and *T. asiatica* genomes.

	Genome coverage	90% confidence interval coverage	High quality SNV count	SNV density (number per Mb)	Ts/Ti ratio	High quality indel count
T. saginata	65	36-99	20,700	122	2.44	1,014
T. asiatica	68	38-105	60,734	362	2.53	2,359

Supplementary Table 8. Proteases encoded by the top 10% of highly transcribed genes.

Gene ID	Classification in MEROPS	FPKM
TASs00168g08721	M41	30.88
TASs00008g02077	M17	1,643.08
TASs00023g03707	S01A	1.33
TASs00002g00521	C01A	112.22
TASs00039g04870	A33	0.00
TASs00007g01894	M20F	29.20
TASs00011g02536	M24B	21.60
TASs00077g06664	M12B	24.87
TASs00007g01772	C12	189.84
TASs00052g05627	A33	0.75
TASs00005g01387	C14A	22.58
TASs00023g03699	M24A	36.20
TASs00009g02152	S09X	26.50
TASs00005g01379	M16B	76.77
TASs00020g03468	A33	8.64
TASs00062g06085	T06	0.00
TASs00039g04869	A33	4.81
TASs00001g00245	M67A	105.07
TASs00026g03980	C19	21.04
TASs00001g00046	T03	5.20
TASs00011g02578	C13	45.52
TASs00067g06288	A33	1.43
TASs00056g05828	M01	123.88
TASs00072g06480	T06	0.05
TASs00012g02728	C50	59.94
TASs00012g02778	C19	13.34
TSAs00039g05585	M16A	32.59
TSAs00067g07425	M12A	95.07
TSAs00002g00832	M17	80.30
TSAs00014g03083	T01A	179.91
TSAs00014g02991	T01A	289.23
TSAs00005g01510	M16C	64.11
TSAs00025g04224	C85B	128.37
TSAs00017g03421	M12A	60.28
TSAs00066g07344	S33	43.11
TSAs00049g06333	C12	94.01
TSAs00012g02793	M67A	125.38
TSAs00003g01170	M28B	380.15
TSAs00059g06971	S26B	154.40
TSAs00051g06463	T01A	82.67

TSAs00040g05646	T02	232.97
TSAs00013g02849	A01A	409.09
TSAs00038g05492	M16B	226.55
TSAs00025g04271	M67A	191.99
TSAs00001g00199	T01A	166.94
TSAs00063g07171	C14A	55.21
TSAs00001g00475	C95	63.52
TSAs00007g01895	C01A	774.25
TSAs00047g06156	C01A	187.34

Supplementary Table 9. Comparison of protease inhibitor prevalence in different species.

Type	*T. saginata*	*T. asiatica*	*E. multicularis*	*S. mansoni*	Target protein
I29	6	7	7	7	cysteine peptidases from family C1
I15	2	2	1	1	serine endopeptidases in family S1
I87	8	8	0	0	HflC (*Escherichia coli*)
I63	1	1	1	2	metallopeptidase pappalysin-1
I08	1	1	1	0	serine and metallo endopeptidases
I01	2	2	3	4	serine endopeptidases（Kunitz inhibitors）
I21	1	1	1	0	serine endopeptidase from family S8
I02	22	20	21	7	serine peptidases（Kunitz inhibitors）
I25A	2	2	1	1	papain-like cysteine peptidases in family C1
I51	1	1	1	1	serine carboxypeptidase Y
I32	2	2	2	3	caspases, cysteine endopeptidases from family C14
I25B	1	1	1	3	papain-like cysteine peptidases in family C1
I39	9	9	2	2	endopeptidases regardless of catalytic type
I04	6	6	5	9	serine peptidases
I93	7	6	0	0	metallopeptidases
I84	0	1	1	0	serine peptidase
I20	0	0	0	1	serine endopeptidase
I71	0	0	0	5	cysteine peptidase

Supplementary Table 10. GO enrichment analysis of *T. asiatica* E/S proteins.

GO ID	GO terms	GO catergory	P-value	Number of genes
GO:0004866	endopeptidase inhibitor activity	F	9.85E-13	17
GO:0061135	endopeptidase regulator activity	F	9.85E-13	17
GO:0005576	extracellular region	C	1.60E-12	20
GO:0030414	peptidase inhibitor activity	F	1.60E-12	17
GO:0061134	peptidase regulator activity	F	1.60E-12	17
GO:0004857	enzyme inhibitor activity	F	4.80E-12	17
GO:0004867	serine-type endopeptidase inhibitor activity	F	3.73E-11	13
GO:0044421	extracellular region part	C	5.29E-06	10
GO:0008233	peptidase activity	F	7.98E-06	25
GO:0005578	proteinaceous extracellular matrix	C	1.24E-04	7
GO:0070011	peptidase activity, acting on L-amino acid peptides	F	1.37E-04	22
GO:0005201	extracellular matrix structural constituent	F	1.44E-04	6
GO:0030246	carbohydrate binding	F	1.50E-04	8
GO:0031012	extracellular matrix	C	2.17E-04	7
GO:0005179	hormone activity	F	3.00E-04	4
GO:0030234	enzyme regulator activity	F	9.49E-04	19
GO:0051213	dioxygenase activity	F	9.66E-04	5
GO:0044420	extracellular matrix part	C	9.66E-04	5
GO:0005581	collagen	C	9.66E-04	5
GO:0031418	L-ascorbic acid binding	F	9.66E-04	4
GO:0031406	carboxylic acid binding	F	9.66E-04	4
GO:0048029	monosaccharide binding	F	9.66E-04	4
GO:0043177	organic acid binding	F	9.66E-04	4
GO:0005102	receptor binding	F	1.05E-03	7
GO:0019842	vitamin binding	F	2.60E-03	4
GO:0015020	glucuronosyltransferase activity	F	2.81E-03	5
GO:0015018	galactosylgalactosylxylosylprotein 3-beta-glucuronosyltransferase activity	F	2.81E-03	5
GO:0006508	proteolysis	P	4.43E-03	24
GO:0016706	oxidoreductase activity	F	4.66E-03	4
GO:0017171	serine hydrolase activity	F	7.46E-03	7
GO:0008236	serine-type peptidase activity	F	7.46E-03	7
GO:0004553	hydrolase activity, hydrolyzing O-glycosyl compounds	F	1.09E-02	7
GO:0016798	hydrolase activity, acting on glycosyl bonds	F	1.92E-02	7
GO:0005506	iron ion binding	F	3.35E-02	5

Supplementary Table 11. GO enrichment analysis of *T. saginata* E/S proteins.

GO ID	GO terms	Category	P-value	Number of genes
GO:0030414	peptidase inhibitor activity	F	1.35E-14	19
O:0061134	peptidase regulator activity	F	1.35E-14	19
GO:0004857	enzyme inhibitor activity	F	3.45E-14	19
GO:0004866	endopeptidase inhibitor activity	F	3.45E-14	18
GO:0061135	endopeptidase regulator activity	F	3.45E-14	18
GO:0004867	serine-type endopeptidase inhibitor activity	F	2.54E-10	13
GO:0005576	extracellular region	C	4.04E-10	18
GO:0030246	carbohydrate binding	F	1.76E-05	9
GO:0008233	peptidase activity	F	1.29E-04	22
GO:0030234	enzyme regulator activity	F	1.43E-04	21
GO:0070011	peptidase activity, acting on L-amino acid peptides	F	1.74E-04	21
GO:0005179	hormone activity	F	2.44E-04	4
GO:0051213	dioxygenase activity	F	1.06E-03	5
GO:0031418	L-ascorbic acid binding	F	1.06E-03	4
GO:0031406	carboxylic acid binding	F	1.06E-03	4
GO:0048029	monosaccharide binding	F	1.06E-03	4
GO:0043177	organic acid binding	F	1.06E-03	4
GO:0005102	receptor binding	F	1.18E-03	7
GO:0019842	vitamin binding	F	2.78E-03	4
GO:0016706	oxidoreductase activity	F	5.63E-03	4
GO:0004175	endopeptidase activity	F	8.27E-03	12
GO:0004553	hydrolase activity, hydrolyzing O-glycosyl compounds	F	8.47E-03	7
GO:0044421	extracellular region part	C	1.41E-02	6
GO:0016798	hydrolase activity, acting on glycosyl bonds	F	1.46E-02	7
GO:0008234	cysteine-type peptidase activity	F	1.76E-02	7
GO:0006508	proteolysis	P	2.79E-02	22
GO:0005506	iron ion binding	F	3.86E-02	5
GO:0017171	serine hydrolase activity	F	4.45E-02	6
GO:0008236	serine-type peptidase activity	F	4.45E-02	6

Supplementary Table 12. *T. asiatica* genes specific to *T. saginata* by strict searches.

Gene ID	location	length of ORF
TASs00005g01300	Scaffold00005(48650,49808,+)	357
TASs03877g13163	Scaffold03877(1,1844,+)	339
TASs00016g03199	Scaffold00016(1126862,1127200,-)	339
TASs00303g09793	Scaffold00303(76562,77393,-)	342
TASs00144g08348	Scaffold00144(22309,23052,+)	465
TASs00021g03624	Scaffold00021(1051430,1051753,-)	627
TASs00429g10448	Scaffold00429(706,1293,+)	303
TASs04711g13246	Scaffold04711(335,1014,+)	627
TASs00323g09921	Scaffold00323(34434,35564,+)	324
TASs00207g09118	Scaffold00207(207,545,+)	339
TASs01193g11994	Scaffold01193(5525,5863,-)	339
TASs00144g08368	Scaffold00144(188145,188453,+)	309
TASs00127g08055	Scaffold00127(244868,247118,+)	375
TASs00073g06507	Scaffold00073(202273,204740,+)	306
TASs00064g06144	Scaffold00064(118247,119023,-)	228

Supplementary Table 13. Datasets used in comparative analyses.

Species	Source	Data version
E. multilocularis	ftp://ftp.sanger.ac.uk	version 3
E. granulosus	ftp://ftp.sanger.ac.uk	version 3
H. microstoma	ftp://ftp.sanger.ac.uk	version 1
S. mansoni	ftp://ftp.ensemblgenomes.org/	release-22
S. japonicum	http://www.chgc.sh.cn/	version 3
A. suum	ftp://ftp.wormbase.org/pub/wormbase/	PRJNA13758
C. elegans	ftp://ftp.wormbase.org/pub/wormbase/	PRJNA80881
G. salaris	http://invitro.titan.uio.no/gyrodactylus/downloads.html	version 1
S.mediterranea	http://parasite.wormbase.org/	SmedGD_c1.3

Supplementary Methods

1. Sample preparation and whole genome sequencing

1.1 Parasite samples

Adult *T. asiatica* and *T. saginata* worms were collected from two patients in Dali, Yunnan Province, China (one worm per species). The adult tapeworms (*T. saginata* and *T. asiatica*) were rinsed five times, 5 min each, at room temperature with PBS containing penicillin (100 U mL^{-1}) and streptomycin (0.1 mg mL^{-1}), and then cultured in sterile Hank's balanced salt solution (HBSS) for one day at 37ºC to further remove host contaminants. Approximately 20,000 eggs isolated from gravid proglottids of the *T. saginata* adult were used to orally infect one 3-month old male calf to prepare tissues for transcriptome analyses. Metacestode tissue of *T. saginata* was retrieved from skeletal muscles of the infected animal 7 weeks after the infection. The calf was housed and cared in according to the Animal Ethics Procedures and Guidelines of the People's Republic of China[1]. All experimental procedures were approved by Institutional Committee for the Care and Use of Experimental Animals, Lanzhou Veterinary Research Institute, Chinese Academy of Agricultural Sciences (No. LVRIAEC2010-002).

1.2 DNA preparation and library construction

Genome DNA was isolated from ~ 50 middle proglottids immediately after the scolex from a single worm for each species without any mature and gravid regions, using Chemagic DNA Tissue Kit (Chemagen). We calculated the frequency distribution of MAF (minor allele frequencies) (see Section 3.3). The clonality was also confirmed by minor allele frequency analysis that were significantly peaked at 0.5 for each genome, which also indicated that the two genomes were diploid (Supplementary Fig. 17). The following DNA libraries were constructed using the standard Illumina protocol (Illumina) at the Beijing Institute of Genomics, Chinese Academy of Science: two paired-end libraries (300 bp and 500 bp) and three mate-paired libraries (1 kb, 5 kb and 10 kb) for *T. saginata*, and two paired-end libraries (500 bp) and seven

mate-paired libraries (2 kb, 2 kb, 3.5 kb, 5 kb, 5 kb, 7 kb and 10 kb) for *T. asiatica*. Paired-end and mate-pair sequencing were performed on the Illumina Genome Analyser IIx (for *T. saginata* DNA libraries) or the Illumina HiSeq 2000 (for *T. asiatica* DNA libraries).

1.3 High-quality read extraction

The quality of raw reads was estimated using the program FastQC[2]. Contaminating reads derived from bacteria or hosts (i.e., humans, pigs and cattle) were filtered by searching the NCBI database (ftp://ftp.ncbi.nlm.nih.gov/genomes/) using in-house Perl scripts. After removing duplicate reads, low quality (cutoff score=20) and contaminating reads, ~ 25 Gb (*T. saginata*) and 21 Gb (*T. asiatica*) high-quality DNA sequences were obtained for subsequent assemblies and analyses.

1.4 Genome size estimations

Genome sizes of *T. saginata* and *T. asiatica* were estimated from *k*-mer frequency distributions. The program Jellyfish (v2.1.3)[3] was applied to count occurrence of 17-mers using reads from paired-end DNA libraries (300 bp and 500 bp libraries for *T. saginata* and two 500 bp libraries for *T. asiatica*). The distributions of 17-mer frequencies were used to calculate the genome sizes with the formula: M=N*(L-K+1)/L and G=T/N, where T represents the total high-quality sequence bases, N represents the actual sequencing depth, L represents the average length of reads, K represents the *k*-mer length (17 bp in this case), M represents the peak in the distribution of the 17-mer frequencies, and G represents the estimated genome size. The estimated genome sizes of *T. saginata* and *T. asiatica* were both at ~ 260 Mb that is much larger than that of *E. multilocularis* (115 Mb)[4] (Supplementary Fig. 18).

2. Genome assembly

2.1 Construction of genome contigs and scaffolds

Genome contigs were constructed from high-quality reads using SOAPdenovo (v1.05)[5] and ABySS (v1.3.5)[6] with a *k*-mer size of 35 and 37 for *T. saginata* and *T. asiatica*, respectively. The

assemblies produced by the two programs were merged using in-house Perl scripts to give the final sets of contigs. Genome scaffolds were constructed from the contigs using program SSPACE (version PREMIUM-2.3)[7] that maps paired-end and/or mate-pair data using Bowtie[8] to pre-assembled contigs and scaffolds with two iterations. Gaps in the scaffolds were closed with 10 iterations of GapFiller (v1-10)[9]. The assemblies were filtered using BLAT[10] self-to-self searches to produce non-redundant scaffolds, resulting in 3,626 (*T. saginata*) and 6,904 (*T. asiatica*) scaffolds with total lengths of 169.1 Mb (*T. saginata*) and 168.7 Mb (*T. asiatica*), respectively (Supplementary Table 1).

2.2 Evaluation of assemblies

The qualities of assemblies were evaluated by contiguity-based statistics (such as N50). The completeness of the assemblies was evaluated using the CEGMA (Core Eukaryotic Genes Mapping Approach) pipeline[11], in which 89.52% (*T. saginata*) and 90.32% (*T. asiatica*) of the 248 core eukaryotic genes were mappable in the two draft genomes. The assembles were also evaluated by mapping with the RNA-seq data using BLAT with default settings[10], in which more than 98% of cDNA sequences (constructed with the *de novo* assembly software Trinity (v2.0.3)[12]) were covered by each assembly (Supplementary Fig. 1).

3. Genome content analysis

3.1 Repeat element identification

Species-specific repeat libraries were constructed using RepeatModeler (v1.0.5) (http://www.repeatmasker.org) with default parameters. The resulting repeat libraries plus the default library from RepeatMasker database (http://www.Repeatmasker.org) were used to detect repeat sequences in the genomes of *T. saginata* and *T. asiatica* by RepeatMasker (v4.0.5,). Tandem repeats were identified using TRF (Tandem Repeat Finder, version trf407b)[13]. Transposable elements (TEs) were identified by homology-based searches against Repbase TE library (http://www.girinst.org/) using RepeatMasker. Long terminal repeat (LTR) transposons were identified with the LTR-Finder v1.0.5[14]. To avoid redundancy, only the longest sequences

from the overlapping matches were selected by Perl scripts. The estimated repeat sequences account for 10.38% (17.5 Mb) and 10.90% (17.3 Mb) in the genome assemblies of *T. saginata* and *T. asiatica*, respectively. Correspondingly, these repeats contained 2.26% and 1.93% retrotransposons (including LTRs, long interspersed nuclear elements (LINE), and short interspersed nuclear elements (SINEs)), 0.07% and 0.21% DNA transposons, 0.87 % and 0.62% simple tandem repeats, and 7.18% and 8.13% unclassified dispersed elements (Supplementary Table 2).

3.2 Non-coding RNA species

The tRNA genes were predicted by tRNAscan-SE (v1.3.1)[15] with general eukaryote parameters, by which 339 and 353 tRNA genes were identified from the *T. saginata* and *T. asciatica* genomes, respectively. Ribosomal RNA (rRNA) genes were identified by searching the genome assemblies against the Rfam database (http://rfam.xfam.org/) using the program rfam_scan.pl[16]. Gene encoding miRNA species were identified by searching the miRNA mature sequences in miRBase (www.mirbase.org) by BLASTN (E-value $<1e^{-10}$ and identity >95%). All hits with an extended 90 nucleotides flanking on each side were used for predicting RNA secondary structures using RNAfold (v2.1.5)[17] with default settings. MiRNA candidates were selected using the following criteria: 1) candidate sequences were present in one of hairpin precursor arms, 2) the minimum free energy for the hairpin structures was -20 kcal mol^{-1}, and 3) hairpins were located in intergenic regions or introns. Totally, 61 (*T. saginata*) and 69 (*T. asiatica*) miRNA species were predicted (Supplementary Table 3).

3.3 Heterozygous single nucleotide variations and indels

The high-quality paired-end reads were mapped to the genome assemblies using Bowtie2[18]. Reads corresponding to PCR duplicates were removed by MarkDuplicates from PICARD (v1.119) (http://picard.sourceforge.net), followed by base quality recalibration and indel realignment by GATK (v3.5)[19] based on a similar coverage of ~65× in each genome. In total, 120,446,124 (*T. asiatica*, 71.40% of assembly) and 126,195,930 (*T. saginata*, 74.63%) "Callable loci" were used

to call SNVs or indels. SNVs and indels were detected by HaplotypeCaller from GATK and filtered as follows: 1. Sites with very high or low coverage (outside the 90% confidence interval of genome coverage) were excluded from further analysis. 2. Sites with high mapping quality score (MQ ≥40) were retained. 3. Heterozygous sites with FS (Phred-scaled p-value using Fisher's exact test to detect strand bias) value higher than 60.0 were removed. 4. Filtered by other parameters in GATK (i.e., QualByDepth < 2.0, MQRankSum < -12.5 and ReadPosRankSum < -8.0). 5. Indels were removed if FS value was > 200.0 or QualByDepth was < 2.0. To ensure the results from the two genomes are comparable, we calculated the proportion of "callable loci" in GATK and the proportion of variants removed by each filter step. After all the filter steps, 32.74% and 28.82% of original heterozygous sites in the *T. asiatica* and the *T. saginata* genomes were retained respectively.

Totally, we identified 60,734 SNVs (504 sites per Mb in callable loci; 362 sites per Mb in assembly) and 2,359 indels in the *T. asiatica* genome and 20,700 SNVs (164 sites per Mb in callable loci; 122 sites per Mb in assembly) and 1,014 indels in the *T. saginata* genome (Supplementary Table 7). As a comparison, we also extracted data with a coverage of ~41× to check whether the result is significantly changed or not in comparison with the case with a coverage ~65 ×. After the same filtering steps, 60,679 SNVs and 2,326 indels, and 20,637 and 1,004 indels were identified in the *T. asiatica* and *T. saginata* genomes, respectively. The result is similar to that based on a ~65 × coverage, indicating that the coverages for SNV detection were saturated and the results were comparable.

Among the SNVs, 6.90% (*T. asiatica*) and 5.96% (*T. saginata*) were located in protein-coding genes, including 769 (*T. saginata*) and 1170 (*T. asiatica*) genes. GO enrichment was carried out on these genes by Blast2GO [20]. Significant enriched GO terms (p<0.01) are presented in Supplementary Data 3 and Supplementary Fig. 14.

4. RNA preparation and sequencing

4.1 RNA preparation and sequencing

Total mRNA was extracted from the metacestode tissue of *T. saginata* and the adult of *T. asiatica* using the TRIzol® Reagent (Invitrogen, USA) and treated with RNase-free DNase (NEB, USA). Then the polyadenylated (polyA+) mRNA was fragmented with 10× Fragment buffer (Ambion, USA). Paired-end libraries (300 bp fragments) were constructed using the fragmented mRNA according to mRNA sequencing sample preparation protocol (Illumina, USA), and sequenced on an Illumina HiSeq 2000 sequencer, at Beijing Institute of Genomics, Chinese Academy of Science.

4.2 Assembly and analysis

After removing low-quality reads and adaptor sequences from the raw sequence reads, two approaches were used to reconstruct transcripts: genome-referenced mapping and *de novo* assembly. In genome-referenced mapping, high-quality RNA-seq reads were mapped to the genomes using TopHat (v2.0.12)[21] and transcripts were built by Cufflinks (v2.0.2)[22] with default settings. In *de novo* assembly, Trinity (v2.0.3) was used to reconstruct transcripts from RNA-seq reads. The transcripts from the two strategies were then merged and aligned to the genome to resolve gene structures using PASA (v2.0.0)[23]. The FPKM values (fragments per kilobase of transcript per million fragments mapped) of gene sets were calculated using Cufflinks (v2.0.2) with -G parameter using the final EVM integrated GFF file as the reference (see below).

5. Gene model prediction and annotation

5.1 Gene prediction

Homology-based, *ab initio* and transcriptome based prediction methods were applied to predict protein-coding genes. 1) **Homology-based gene prediction**. All protein sequences from the Swiss-Prot database (http://www.uniprot.org/) and protein sequences of the previously sequenced helminth genomes (i.e., *T. solium*, *Echinococcus granulosus*, *E. multilocularis*, *Hymenolepis microstoma*, *Schistosoma japonicum*, *S. mansoni*, *Ascaris suum* and *Caenorhabditis elegans*) were aligned to the assemblies of *T. saginata* and *T. asiatica* using the program SPALN (v2.0.6)[24] with default settings. The alignments were extended by 1 kb for each side of the hits to identify

start and (or) stop codons. 2) ***Ab initio* gene prediction.** Four *ab initio* gene prediction programs, including Augustus (v3.0.1)[25], Genemark (v2.3)[26], GlimmerM (v3.0.2)[27] and SNAP (v2013-11-29)[28] programs, were employed to predict genes in repeat-masked genomes. High-quality training sets for *ab initio* gene predictors were constructed using high-confidence gene models extracted from the reconstructed transcripts based on RNA-Seq data using PASA (v2.0.0). 3) **Transcriptome-based gene prediction**. RNA-Seq paired-end reads were mapped to the genomes using TopHat (v2.0.12), and the alignments were used to construct transcripts by Cufflinks (v2.0.2). The non-redundant and full-length transcripts were aligned to the genomes to determine the exon and intron locations using PASA. Finally, a set of weighted consensus gene structures from the three predictions were generated using the software EVidenceModeler (EVM, v1.1.1)[23]. PASA was used to add the UTR annotations to the existing gene structures. In total, 13,161 and 13,323 protein-encoding genes were generated for *T. saginata* and *T. asiatica* genomes, respectively.

5.2 Gene functions

The function annotations of genes were performed by searching the predicted proteins against public database using BLASTP (e-value <1e^{-5}), including NCBI non-redundant (nr) protein database, Uniprot (http://www.uniprot.org/) and the KEGG (Kyoto Encyclopedia of Genes and Genomes) database (http://www.genome.jp/kegg/). InterproScan5[29] was used to identify known domains, assign GO terms and classify function annotations for the predicted proteins.

5.3 Gene structures

We observed a bimodal distribution of short introns (i.e., ~36 bp for peak-1 and ~73 bp for peak-2) (Fig. 1) in both the *T. saginata* and *T. asiatica* genomes. This feature was also observed in other sequenced tapeworms and an ectoparastic neodermata, *Gyrodactylus salaries*[30], albeit with small size variations, but absent in flukes, which suggests that this is an ancient feature shared by parasitic tapeworms. In the *T. asiatica* genome, 1,068 and 1,498 genes contain peak-1 (34-35 bp) and peak-2 (73-74 bp) introns, respectively. In the *T. saginata* genome, 1,106 and

1,436 genes contain peak-1 (36-37 bp) and peak-2 (73-74 bp) introns, respectively. The Wilcoxon rank sum test (two-sided) was used to compare the intron numbers of the two datasets in each genome (Supplementary Fig. 3). The GO enrichment analyses for the genes containing peak-1 and peak-2 introns were performed by BLAST2GO[20] (Supplementary Data 1). As the splicing machinery is limited in its ability to recognize introns of a certain length[31], these introns are probably under evolutionary selection to remain short (Fig. 1; Supplementary Fig. 4-5).

Gene models of endo-parasitic flatworms *T. asiatica, T. saginata, E. multilocularis, H. microstoma, Schistosoma mansoni, Schistosoma japonicum* and *Clonorchis sinensis* in Wormbase (http://parasite.wormbase.org), an ectoparasitic flatworm *G. salaries* (http://invitro.titan.uio.no/gyrodactylus/), and two outgroup species (*Caenorhabditis elegans* and *Drosophila melanogaster*) (http://ensembl.org) were used to compare the exon and intron features. Exon-intron structures of transcripts assembled from the RNA-seq data, using the pipeline similar to section 4.2 (Cufflinks and Tophat), for *T. asiatica*, *E. multilocularis* (ERR1328265 and ERR1328267) and *S. mansoni* (ERR233398) were also involved in the analysis of exon and intron features. A striking feature in the parasitic flatworms that is supported by evidence from both the gene models and expressed transcripts is the apparent preference of the mean lengths of neighboring introns and exons. The minimal mean lengths of intron pairs flanking small exons that share a specific length (<400 bp) are 502 bp (*T. saginata*) and 370 bp (*T. asiatica*) long, while that for *Schistosoma* is 1,204 bp (Fig. 1d and supplementary Fig. 4-5), implying certain species-dependent length requirement for exon-intron recognition. This feature also appears in *Caenorhabditis elegans* and *Drosophila melanogaster* (Supplementary Fig. 5).

6. Homolog comparison and collinearity detection

6.1 Homolog comparison

The predicted proteins of *T. asiatica* were compared with those of other tapeworms (i.e., *T. saginata, T. solium, E. multilocularis* and *H. microstoma*) (Fig. 2) by BLASTP (E-value <1e^{-4}). A more restricted search for *T. asiatica*-specific genes was performed by TBLASTN (E-value <1e^{-3})

against the *T. solium* and *T. saginata* assemblies (Supplementary Table 12). Gene expressions were evaluated by RNA-seq data. Candidate genes for differential molecular diagnosis between *T. asiatica* and *T. saginata* were identified from single-copy genes derived from the OrthoMCL[32] analysis (see S7 section) and ranked by synonymous substitutions per synonymous site (K_s) values calculated using Codeml (CodonFreq = 2, runmode = -2) implemented in PAML package (v4.8)[33]. The top 100 pairs of genes were retained as potential molecular markers (Supplementary Data 13).

6.2 Synteny and collinearity

For comparative analysis, the assemblies of *T. saginata*, *T. asiatica* and *T. solium* were aligned using nucmer (setting: -mum) implemented in MUMmer (v3.0)[34], followed by delta_filter program (setting: -r and -q) in conjunction for a one-to-one mapping between the references and queries. Altogether, the alignments between *T. saginata* and *T. asiatica* scaffolds showed high similarities at the nucleotide level. Around 138 Mb (~81%) sequences from each species could be directly aligned by one-to-one matches (mean length = 5.4 kb) with an overall 92.26% identity and limited structural variability. Much less genomic syntenic regions were observed between the *T. solium* scaffolds and those of *T. saginata* (108 Mb) or *T. asiatica* (107 Mb) (overall identity = 88.53%, mean length = 3.8 kb). Nearly all the syntenic breaks between their genomes occurred at the ends of scaffolds, suggesting that true synteny is higher between the two tapeworm genomes. Collinearity for the orthorlogue gene blocks on the scaffolds was analyzed using software MCscanX[35] with default parameters, which also revealed a significantly higher synteny between *T. saginata* and *T. asiatica* (n1=7201; n2 =7,212; 292 blocks) than that between *T. asisatica* and *T. solium* (6,055; 6,058; 303).

7. Gene family analysis

7.1 Identification of gene families

To analyze gene families, the protein sequences from ten flatworms and roundworms (i.e., *T. saginata*, *T. asiatica*, *T. solium*, *E. granulosus*, *E. multilocularis*, *H. microstoma*, *S. japonicum*, *S.*

mansoni, *A. suum* and *C. elegans*) were collected (Supplementary Table 13), and those proteins with length ≥30 aa were used to calculate pair-wise similarities using BLASTP (E-value <=1e^{-5}). The gene families were constructed based on the similarities from the BLASTP results using the software OrthoMCL (v2.0.9)[32] with an inflation of 1.5 (seen section 7.2) for clustering groups. A total of 19,621 families were clustered for the 130,868 proteins. There were 5,888 protein families shared by all species within the Taeniidae lineage (Supplementary Fig. 19).

The HSP70 family is known to be highly expanded in tapeworms[4]. However, the expansion is much greater in *T. saginata* (n=96) and *T. asiatica* (n=80) than those in other tapeworms (~22-32) (Supplementary Fig. 9). A significant number of HSP70 proteins contain signal peptides but no transmembrane domains, indicating that they are secreted proteins and may be involved in host-parasite interaction. Several additional protein families are also more greatly expanded in beef and Asian tapeworms than in other related species, including dynein motor protein, ubiquitin-conjugating enzyme, retinal guanylyl cyclase 2, peptidase family M1, galactosyltransferase and polycystin. Additionally, more NK-2 homebox genes are present in *T. saginata* (n=12) and *T. asiatica* (n=9), than those in *E. multilocularis* (n=1), *E. granulosus* (n=1) and *S. mansoni* (n=0). Interestingly, the number of members of this gene family among these parasites is likely to correlate with their general proglottid numbers.

7.2 Evaluation of the inflation parameter in OrthoMCL

To assess the robustness of group assignments in association with the inflation parameter in orthoMCL, we investigated the accuracy of the clustering results by examining the consistency of the groups with respect to enzyme commission (EC) numbers assigned by KEGG, reasoning that EC numbers are probably among the most reliable functional assignments that have been widely applied during genome annotation. The accuracy of the clustering results of proteins for which a complete EC number has been assigned (EC-annotated sequences) was investigated by adjusting I values (I = 1.2, 1.5, 2, 2.5, 3, 3.5 and 4), according to the ref.[32]. The complete data sets involved in the assessments included a total of 1,557 and 1,529 EC-annotated sequences in the *T. saginata*

and *E. multilocularis* genomes respectively. For each dataset, the sensitivity, specificity and accuracy were evaluated by making statistics of true/false positive and true/false negative numbers in the orthoMCL groups with EC assignments, using the same equations as described in [36]. According to the results, the inflation value of 1.5 appeared to best balance sensitivity and specificity, consistent to the evaluation result by [32], resulting with the maximum accuracy value for both the two tapeworms (Supplementary Fig. 20). Therefore, we used the inflation value of 1.5 in orthoMCL for comparatively functional analysis of gene families in the tapeworms.

7.3 Phylogenetic reconstructions

A total of 747 single-copy genes were extracted from the flatworms and roundworms for phylogenetic reconstructions (see section 7.1). Individual protein alignments were performed using ClustalW2 (v2.1)[37]. Corresponding CDS (nucleotide sequences) were also individually aligned. Both protein and nucleotide alignments were concatenated. Gaps were removed by program Trimal (v1.4)[38]. The best evolution models for the protein and CDS alignments were estimated using ProtTest (v3.4)[39] and ModelTest (v2.1.4)[40], respectively. Maximum likelihood trees were reconstructed with 200 bootstrapping replicates using RAxML (v8.0.24)[41] with the best fit models with the consideration of fraction of invariance (I) and a 4-rate gamma (G) heterogeneity (i.e., LG+I+G+F for proteins and GTR+I+G for CDS) (Supplementary Fig. 13).

7.4 Gene family size analysis

Gene family sizes were inferred from the gene family profile obtained by the program OrthoMCL. The program Dollop implemented in PHYLIP package (v3.695)[42] was used to estimate the minimal gene set of different ancestral nodes by Dollo and polymorphism parsimony methods with two states (0 and 1) to determine gene family gain or loss at branches since divergent from their parent nodes. There are 8,179 and 8,183 gene families in the *T. saginata* and *T. asiatica* genomes, respectively (Fig. 4a), and 5,385 ancestral families in the ancestral platyhelminth lineage. The program Café (v3.0)[43] was used to estimate the most likely gene number for each family at internal nodes in the phylogenetic tree.

8. Divergence dates and gene duplications (GDs)

8.1 Divergence time and mutation rate estimations

Divergence times were estimated from a refined concatenated CDS alignment containing 102 single copy genes without partitions by BEAST2 (v2.1.3)[44] with a relaxed log normal model that were calibrated with two previously estimated dates between *Ascaris* and *Caenorhabditis* (260 Mya)[45] and between Platyhelminth and Nematoda (632 Mya)[46]. The best RAxML tree (Section 7.2) was used as the starting tree. The general time reversible (GTR) substitution model with an estimated gamma distribution of substitution rates was selected with jModeltest. Priors used calibrated Yule model, time calibration constraints as described above, and default settings for other priors. Samples from the posterior were drawn every 1,000 steps over a total of 10,000,000 steps per MCMC run. The convergence of likelihood values was determined by Tracer (v1.6). Trees were annotated by TreeAnnotator (v2.1.2) using maximum clade credibility tree and median heights settings with 25% burnin. The calculated split time between the flukes and the tapeworms (319.66 Mya) agreed with the recently reported 270 million-year-old fossilized tapeworm eggs in shark coprolite. Our analysis suggested that *T. asiatica* diverged from *T. saginata* at 1.14 Mya (95% highest probability density: 0.5479-1.4292). The mutation rates in the *T. asiatica* and *T. saginata* genomes were estimated by dividing the branch lengths with the divergence time (i.e., 1.14 Mya between the beef and Asian tapeworms) (Supplementary Fig. 12).

8.2 Relative evolutionary ratio test

We used the 747 single-copy orthologue genes shared by the six tapeworms to estimate the nucleotide substitution rates. The Tajima's Relative Rate Test was performed using DAMBE (v5.5.21)[47] to test the rate-constancy hypothesis (null) between the lineages *T. asiatica* and *T. saginata* using *T. solium* as outgroup, for both synonymous substitution and nonsynonymous substitutions (p<0.01). The evolution rates of protein-coding genes in *T. asiatica* (0.00467 mutation per site per site) were found to be much higher than that of *T. saginata* (0.003788 mutation per site per site).

8.3 Paralogous group reconstructions

We employed a Blast-based method to construct paralogous groups in each genome for synonymous substitutions per synonymous site (K_s) distribution analysis. The analysis was performed as follows: An all-against-all protein sequence similarity search was performed by using BLASTP (e-value ≤ $1e^{-10}$, identity score ≥ 30%), followed by clustering the paralogous groups using Markov Clustering (MCL)[48] (mclblastline pipeline) (–mcl-I = 2.0, with other settings default).

In order to estimate the robustness of clustering of paralogous groups with respect to different inflation values of MCL method, we evaluated a series of inflation values (I =1.5, 1.8, 2, 2.5 and 3) in the mclblastline pipeline by manual assessments. In the end, the clusters generated with a inflation index of 2.0 in mclblastline pipeline (–mcl-I = 2.0) that may represent a slightly lower cluster tightness (I value) than the expected value for exactly clustering paralogous groups in the species were used for further analyses, to obtain proper paralogous pairs for K_s estimations as more as possible. Furthermore, in order to exclude unreasonable K_s estimations from the false positive paralogous relationships produced from MCL cluttering with a inflation value of 2.0, paralogous gene families were subdivided into subfamilies for which K_s estimates between genes did not exceed a value of 5.0 by an average linkage clustering approach using K_s as a distance measure (as described in ref.[49]) (See Section 8.4). Only K_s estimates lower than 5 were considered in the construction of empirical age distributions[50].

Moreover, whichever of the above inflation values was evaluated, no significant differences between their distributions of K_s values were observed in our analysis. The above inflation values would not differentially affect the subsequent conclusions in our study.

8.4 K_s distribution of paralogous genes

For each paralogous gene group, a protein alignment was constructed using MAFFT (--auto) (v7.147b)[51]. This alignment was used as a guide for aligning the DNA sequences of gene family pairs, using ParaAT (v1.0)[52]. Paralogous gene pairs were retained if the two sequences were

alignable over a length of more than 150 amino acids.

The K_s values of each paralogous gene pair in each genome (*T. saginata*, *T. asiatica*, *T. solium*, *E. multilocularis* and *S. mansoni*) were calculated using the program codeml (CodonFreq = 2, runmodel = -2) in the PAML package (v4.8). Only K_s values ≤ 5 were retained for further analysis. For each pairwise comparison, K_s estimation was repeated five times to avoid suboptimal estimates because the program might fail to find the global maximum likelihood. Large families were subdivided into subfamilies for which K_s values between genes did not exceed a value of 5.0. An average linkage clustering approach was used to correct the redundancy of K_s values (a gene family of n members produces n [n–1]/2 pair-wise K_s estimates for n-1 retained duplication events), as described in ref.[49,50,53]. Briefly, for each family, a tentative phylogenetic tree was constructed by average linkage hierarchical clustering, using K_s as a distance measure. For each split in the resulting tree, corresponding to a duplication event, all m K_s estimates between the two child clades were added to the K_s distribution with a weight 1/m, so that the weights of all K_s estimates for a single duplication event sum up to one. The K_s distributions of gene duplicates in all the highly divergent tapeworms are typically quasi-exponential L-shaped (Fig. 3 and Supplementary Fig. 8).

8.5 K_s distribution of orthologous genes

In order to estimate the divergence of different parasitic flatworms, K_s values of orthologous genes were calculated. The pair-wise orthologous gene relationships were determined by OrthoMCL (see Section 7.1). K_s value for each orthologous gene pair among *T. saginata*, *T. asiatica*, *T. solium*, *E. granulosus*, *E. multilocularis*, *S. japonicum* and *S. mansoni* was calculated according to the same pepiline in section 8.4 (by MAFFT, ParaAT and Codeml) with the same settings. The K_s distribution is shown in Fig. 3, in which a distribution peak for orthologous genes is associated with a speciation event.

8.6 Extensively duplicated genes

In this study, we analyzed the duplications of each protein group after the split of Cestoda and

Trematoda (K_s ≤2.6). We observed that several functional homologous groups (e.g., the surface antigens, HSP70, ubiquitin conjugating enzyme, ryanodine receptor 44f, ankyrin and zinc finger proteins) experienced temporally continuous/extensive duplications, during evolution histories of the tapeworm lineage. These homologous groups are presented in Supplementary Table 5. Among them, the surface antigens (Taeniidae antigens and diagnostic antigen *gp50*) may be of great importance in the survival and/or adaptations to environments, given their interactions with the host immune systems.

We conducted phylogenetic analyses of the protein sequences of *diagnostic antigen gp50* genes of *T. saginata*, *T. asiatica*, *T. solium*, *E. multilocularis* and *H. microstoma*. Sequences less than 150aa were excluded. In total, 191 sequences were used for further analysis. These sequences were aligned using MAFFT (v7.147b) (--auto), and the alignments were optimized by MaxAlign server (http://www.cbs.dtu.dk/services/MaxAlign/). The remained sequences (183 sequences) were re-aligned using MAFFT (v7.147b) (--auto), and the conserved blocks from the multiple alignments were selected by Gblocks server (http://molevol.cmima.csic.es/castresana/Gblocks_server.html) (with a less stringent option) for phylogenetic analysis. The best evolution model for the protein alignments were estimated using ProtTest (v3.4)[39]. Maximum likelihood tree was built with 100 bootstrapping replicates using RAxML (JTT+G+I). The conserved region logo of *Diagnostic antigen gp50* was generated by WebLogo (default settings, http://weblogo.berkeley.edu/). The tree was showed in Fig. 3.

8.7 Gene duplication mode analysis

We used the duplicate_gene_classifier (-s 3 -m 30) algorithm implemented in the MCScanX program to determine possible origination of the duplicate genes for the *T. saginata*, *T. asiatica*, *T. solium* and *E. multilocularis* genomes. According to the classification in the software, origins of the duplicate genes of each genome are classified into whole genome /segmental (i.e. collinear genes in collinear blocks), tandem (consecutive repeat), proximal (in nearby chromosomal region but not adjacent) or dispersed (other modes than segmental, tandem and proximal) duplications.

These GD events are mostly derived from small-scale gene duplications (SSGDs), predominated by dispersed duplications (e.g., 74.14% in *T. asiatica*, 71.95% in *T. saginata* and 69.94% in *E. multilocularis*), followed by tandem (15.69%, 16.69% and 15.08%) and proximal duplications (8.90%, 10.75% and 11.29%) (Supplementary Table 4) in the current assemblies.

8.8 In-paralogous genes identification

Newly duplicated genes (in-paralogous genes) after the divergence of *T. asiatica* and *T. saginata* were determined by InParanoid (v4.1)[54] with default settings. Both *T. asiatica* and *T. saginata* genomes possess a large number of recently duplicated genes (involving 1,075 and 866 in-paralogs after their divergence) that were derived from 614 and 481 duplicate events along each lineage, respectively. Enrichment analysis of the species-specific genes in each of the two tapeworm genomes was performed by Blast2GO, using Fisher's Exact Test (two-sided). Significant enriched GO terms (p <0.01) are presented in Supplementary Table 6. We also compared the gene expression levels (FPKM value) of in-paralogous gene pairs (Supplementary Fig. 11).

Using out-paralogous genes from *T. solium* as outgroups, we compared the evolutionary rate for each pair of the in-paralogous genes arising after the divergence of *T. saginata* and *T. asiatica* by Tajima's Relative Rate Test in DAMBE (v5.5.21)[47]. A number of newly duplicated genes evolve significantly asymmetrically between paralogous pairs (72/804 in *T. asiatica*; 88/592 in *T. saginata*, p <0.05) (Supplementary Data 2).

The phylogenetic trees of fatty acid desaturase (FADS) and low-density lipoprotein receptor (LDLR) sequences were constructed using the method in section 8.6. The best evolution models for the protein alignments of FADS and LDLR were Blosum62+I and JTT+I+G, respectively. These trees were edited by EVOLVIEW (http://www.evolgenius.info/evolview/) (Fig. 4).

FADS and LDLR were expanded along the *T. asiatica* lineage. Each phylogeny of the two families was determined with an overall p-value 0.001 based on a Monte Carlo re-sampling procedure implemented in the software CAFÉ (see Section 7.4), implying the very high

probability of the gene families with the observed sizes among taxa. The branch-specific p-values were obtained for LDLR (0.01) and FADS (0.035) genes in *T. asiatica* by the Viterbi method with the randomly generated likelihood distribution, smaller than the p-value cutoff 0.05. The low p-value indicates a rapidly evolving branch along *T. asiatica* in each family.

8.9 Gene duplication rate comparison

To make assessments of observed gene duplication rates (GDR) in the tapeworms, we performed several additional analyses for comparison as follows:

First, we selected several species (*Caenorhabditis remanei*, Scaffold N50=435kb) (http://parasite.wormbase.org/) (*Anopheles sinensis*, 579 Kb; *A. merus*, 342 kb) (https://www.vectorbase.org) with similar assembly qualities for GDR estimations. The observed GDRs were estimated from the abundance of the very youngest pairs by a method using synonymous site divergence as a proxy for the age of duplicated genes as described in the ref.[55]. To exclude the high statistical uncertainty associated with large estimates of *Ks*, we confined our analyses of *Ks* <0.01. The paralogous gene pairs and gene duplicates were estimated using the method as described in sections 8.3 (--mcl-I=2.0) and 8.4, respectively. In addition, we also calculated the GDR in the *C. briggsae* (Scaffold N50 = 17.4 Mb) genome (http://parasite.wormbase.org/) that represents an assembly of high quality for comparison. For the *Caenorhabditis* species, using a rate of silent-site substitution of 15.6 per site per billion years (BY)[55,56], the estimated rates of origin of new duplicates were 0.0234 and 0.0207 duplicates per gene per million years for *C. briggsae* and *C. remanei*, respectively. The rates are similar to that of *C. elegans* (0.0208) obtained in the ref.[55]. For the *Anopheles* species, we inferred an average rate of silent-site substitution of 1.9 per site per BY by a silent nucleotide site molecular clock calibrated with the *Anopheles* divergence dates from the ref.[57]. The GDRs of *A. sinensis* and *A. merus* were appropriately 0.0046 and 0.0035 duplicates per gene per million years, respectively, similar to a rate of 0.00312 in *Anopheles* genomes estimated via an independent method[57]. The GDRs of the two tapeworms (0.0321 and 0.0404 duplicates per gene per million years for *T.*

saginata and *T. asiatica*, respectively) are remarkably higher than those in the *Anopheles* species and at the same order-of-magnitude with those in the *Caenorhabditis* species.

Second, comparison with a well-assembled tapeworm genome: In order to test whether the phenomenon of high gene duplication rate is shared across the tapeworm lineage, we analyzed the GDR in the well-curated *E. multilocularis* (also from the family Taeniidae) genome, using the *Ks*-based method as described above. This genome has been subject to iterative improvement and represents the highest quality of assembly (N50 = 13.8 Mb) among the currently sequenced tapeworms[4]. An average rate of silent-site substitution of 10.2 per site per BY in the *Echinococcus* species inferred from the divergence rates in section 8.1 was used for calculation. The *E. multilocularis* genome showed an observed rate of 0.0304 duplicates per gene per million years, remarkably similar to those in the *T. asiatica* and *T. saginata* genomes. This evidence suggests that duplicate genes are likely to arise at a similar rate in the tapeworm lineage.

In addition, to estimate the assembly quality of the duplicated genes in the *T. saginata* and *T. asiatica* genomes, we also conducted the following analyses:

First, assembly quality estimation: To assess the assembly quality of the duplicated gene regions (involved in the calculation of the duplication rate) in the two assemblies, we estimated the sequencing coverage for each gene, using high-quality reads. The high-quality paired-end reads were mapped to the genome assemblies using Bowtie2 (v2.2.3), and the coverage of each position in the two genomes was estimated by Samtools (v0.1.19). Most of these genes were well supported by high coverage folds, except for only ~123 (accounting for 11.44%) and 83 (9.58%) genes with coverage folds lower than the half of the average in *T. asiatica* and *T. saginata*, respectively. Furthermore, we also re-checked the sequencing coverages of the regions flanking genes and found no significant differences in coverage distributions between the genes and their adjacent regions (Wilcoxon rank sum test, p-value =0.4661, two-sided). Furthermore, although the coding sequences are highly conserved (98.22% and 96.41%), the overall identities of genes containing introns and flanking regions of these duplicated genes were estimated to be approximately 83.26% and 73.07% in *T. asiatica* and *T. saginata*, respectively, suggesting that these genes have substantially diverged. This evidence implies that most of the involved genes are well assembled.

Second, gene completeness assessment: In order to exclude the possibility that one gene can be wrongly fragmented into two or more gene segments, we estimate the completeness of the paralogous genes in the two assemblies. Altogether, 97.26% and 95.10% gene pairs have overlap regions covering at least 80% of their coding sequences in the alignments in the *T. asiatica* and *T. saginata* genomes, respectively. No gene pair showing an overlap region covering < 50% of their sequences were included in the duplication rate calculation.

Third, as a comparison, we re-calculated the gene duplication rates by using different combinations of cutoffs of the above assessments. Although the rates varied slightly, the order-of-magnitude was not changed (e.g., approximately 0.0267 and 0.0326 corresponding to methods by filtering genes with cutoffs of 50% coverage and 80% completeness in the *T. saginata* and *T. sciatica*, respectively). Varying the cutoffs within reasonable values do not significantly affect the conclusion in this study.

9. Prediction of positively selected genes

One-to-one orthologuous genes were identified from the genomes of six tapeworms (*T. saginata*, *T. asiatica*, *T. solium*, *E. granulosus*, *E. multilocularis* and *H. microstoma*) using programs Inparanoid (v4.0)[54] and Multiparanoid (v1.0)[58]. Only transcripts containing intact coding regions (CDSs) with lengths >100 bp and multiples of three were used in analysis. Individual *T. asiatica* or *T. saginata* genes and their orthologs in other tapeworms were examined for evidence of an in-paralog (a paralog arising from a recent duplication) with respect to other species. Specifically, if either a gene had in-paralogs, then that gene was considered recently duplicated and was excluded from the analysis of positive selection. The removal of a duplicated gene did not require an ortholog set to be discarded entirely, provided both the *T. asiatica* and *T. saginata* genes and ≥ 1 other tapeworm orthologs still remained. These procedures resulted in 6,581 one-to-one orthologous gene groups for further analysis. Multiple protein-coding codon alignments were generated using ParaAT (v1.0)[52] and MAFFT (v7.147b)[51], with default settings. All gaps in the alignments were deleted. The likelihood ratio test (LRT) for selection ($p< 0.05$) on any branch of the phylogeny was performed based on the likelihood values from the Codeml program with

modified Branch-site model A (model= 2, NSsites= 2) as implemented in the PAML package (v4.8). This compared the alternative hypothesis with ω2 estimated (fix_omega = 0 and initial omega = 1.5) with the corresponding null model with ω2 = 1 fixed (fix_omega = 1 and omega = 1) for the lineage *T. asiatica* or *T. saginata* (the foreground branch), respectively. The log likelihood values from alternative (lnL1) and null hypothesis (lnL0) were used to construct the LRT: 2× (lnL1 - lnL0). For the lineage specific LRTs, p-values were computed assuming the null distribution was a 50:50 mixture of a χ^2 (df =1) distribution and a point mass at zero. The method of Benjamini and Hochberg[59] was used to estimate the appropriate p-value threshold for a false discovery rate (FDR) (FDR <0.05). If significant, sites under positive selection determined by Bayes Empirical Bayes (BEB) were reported. Altogether, 134 and 102 genes were identified as positively selected genes (PSG) in *T. asiatica* and *T. saginata*, respectively (FDR< 0.05, LRT) (Supplementary Data 4).

10. Excretory/Secretory (E/S) protein prediction pipeline

A refined bioinformatics workflow was designed to predict ES proteins in the genomes of *T. saginata* and *T. asiatica* (Supplementary Fig. 16), using a strategy of integrating several tools. The algorithm TMHMM (v2.0)[60] was used to predict transmembrane (TM) domains. For the proteins containing only one TM domain, further TM prediction was performed using the Phobius algorithm[61] to discriminate putative TM topologies from signal peptides. All proteins predicted to have a TM domain were discarded for further E/S analysis. SignalP (v4.1)[62] was used to predict signal peptides of classical secretory proteins from the first 70 N-terminal amino acids of each proteins (parameters for eukaryotes and default D-cutoff values). The non-classical secretory proteins were predicted using SecretomeP (v2.0)[63], filtered by NN-scores larger than 0.9 and default options for mammalian organisms. All classical and non-classical secretory proteins were merged together and scanned by TargetP (v1.1)[64] to predict the subcellular localization of mitochondrial proteins (specificity of 90% and default options for non-plant organisms) that were excluded from the E/S protein dataset. The predicted E/S proteins were subsequently scanned for ER targeting signals by PS-Scan[65] (Prosite pattern: PS00014) and

GPI-anchor signals by PreGPI[66] with default parameters. GO terms enrichment of E/S proteins was performed by BLAST2GO by Fisher's Exact Test (two-sided) with Multiple Testing Correction of FDR (FDR <0.05), using the entire proteome as a reference group (Supplementary Tables 10 and 11).

11. Drug target identification

11.1 Proteases and protease inhibitors

Putative proteases and protease inhibitors were detected and classified using the MEROPS batch BLAST server (cutoff: $1e^{-4}$)[67]. If a protein sequence matches members of a peptidase family but lacks any of the expected catalytic residues, it is annotated as a non-protease homolog. Using the approach, we predicted 157 and 161 proteases as well as 142 and 155 non-protease homologs, in the *T. saginata* and *T. asiatica* genomes, respectively (Supplementary Data 5 and 6). These proteases belong to five major classes (aspartic, cysteine, metallo-, serine and threonine), with the metallo- (n=48 in *T. saginata* and 46 in *T. asiatica*), cysteine (n=41 and 44) and serine proteases (n=27 and 30) predominating. Of them, 20 (12.7%; 12.4%) proteases were predicted as classical secretory proteins, including aspartic (n=1), cysteine (n=6), metallo- (n=6), serine (n=6) and threonine (n=1) proteases. Many homologs of these secreted proteins have been considered to participate in host-parasite interactions in other helminthes. Only one secreted aspartic protease, which is a cathepsin D-like protein (A01), can be found in all tapeworms (Supplementary Data 5), whereas more than 10 secreted aspartic proteases exist in the flukes *S. mansoni* and *S. japonicum*. Based on results of the birth and death analysis of gene family (data not shown), we found that *T. saginata*, *T. asiatica* and other tapeworms have a significant loss of A01A family members, rather than the gene expansion seen in flukes. Proteins in the A01 family are typically associated with the external digestion of proteins in mammals, and in the flukes, these proteins have been detected in the guts for digesting host serum[68]. Therefore, the difference in this family between the two parasite classes may be associated with different mechanisms of nutrient acquisition and the loss of the alimentary canal in tapeworms during evolution. In addition, we also identified 71

and 70 natural protease inhibitors (PIs) in *T. saginata* and *T. asiatica*, respectively (Supplementary Data 7 and Supplementary Table 9), belonging to 16 families of serine, cysteine, and metallo-protease inhibitors, with the apparent absence of aspartic (like I33 aspin) and threonine protease inhibitors.

11.2 Identification of G protein coupled receptors (GPCRs).

Phobius was used to identify transmembrane (TM) domains in the predicted *T. asiatica* and *T. saginata* proteomes (filtered by amino acids >250). The resulting proteins with ≥3 and ≤15 TM domains (data set 1) were retained and further searched using HMMER (v3.1b1)[69] with HMMs of GPCRs in the Pfam database (http://pfam.xfam.org/). GPCRs annotated in other tapeworms[4] were also used as queries against the data set 1 by BLASTP search ($1e^{-4}$). The two resulting hits were merged to be further filtered by BLAST search against NCBI ‘nr’ database. The proteins with significant match to un-related proteins were discarded (Supplementary Data 8).

11.3 Kinase classification.

Protein kinase domain-containing genes were extracted from the InterProScan domain annotations. The corresponding domains were clustered with a reference dataset (Human, fly and *C. elegans*) of protein kinase-domains (KINBASE, http://kinase.com/kinbase/FastaFiles/) using OrthoMCL. The domains that failed to be assigned during the clustering were further used to search kinases of other tapeworms for classification[4], using BLASTP (E-value $<1e^{-4}$). The two resulting classifications were combined (Supplementary Data 9).

11.4 Identification of ligand-gated ion channel (LGIC).

LGIC database subunits (http://www.ebi.ac.uk/compneur-srv/LGICdb/) and platyhelminth (*E. multilocularis* and *S. mansoni*)[4,70] LGICs were used as BLAST queries against the predicted tapeworm proteomes to identify LGICs. The resulting hits were used as BLASTP queries against the NCBI ‘nr’ database and proteins displaying homology to unrelated proteins were discarded. Proteins that display LGIC-related homology were retained along with those having no hits in nr

database as the putative LGICs (Supplementary Data 10).

11.5 Analysis of potential drug targets

In order to identify specific drug target proteins in tapeworms that have no sequence significant sequence similarities to those of hosts, we performed homology searches by BLASTP algorithm ($e < 1e^{-4}$) using the potential drug targets proteins, including GPCRS, LGICs, proteases, PIs, kinases, and E/S proteins, as queries against the proteome of humans (http://www.ensembl.org/index.html) (Supplementary Data 11). Proteins that are not homologous to the host proteome were further screened for sequence similarities against known drug targets (Supplementary Data 12). Drug target sequences were extracted before October, 2014 from the following databases: 1. ChEMBL (ftp://ftp.ebi.ac.uk/pub/databases/chembl/DrugEBIlity/releases/3.0/): 16072 drug target protein sequences and 212919 domain sequences; 2. DrugBank (http://www.drugbank.ca/): 3789 proteins; and 3. Therapeutic Targets Database (http://bidd.nus.edu.sg/group/ttd/): 1973 proteins.

Supplementary references

1 The Ministry of Science and Technology of the People's Republic of China. Guidance Suggestions for the Care and Use of Laboratory Animals (2006).

2 Andrews, S. FastQC: A quality control tool for high throughput sequence data. http://www.bioinformatics.babraham.ac.uk/projects/fastqc/ (2010).

3 Marcais, G. & Kingsford, C. A fast, lock-free approach for efficient parallel counting of occurrences of k-mers. *Bioinformatics* **27**, 764-770 (2011).

4 Tsai, I. J. *et al.* The genomes of four tapeworm species reveal adaptations to parasitism. *Nature* **496**, 57-63 (2013).

5 Li, R. Q. *et al.* De novo assembly of human genomes with massively parallel short read sequencing. *Genome Res* **20**, 265-272 (2010).

6 Simpson, J. T. *et al.* ABySS: A parallel assembler for short read sequence data. *Genome Res* **19**, 1117-1123 (2009).

7 Boetzer, M., Henkel, C. V., Jansen, H. J., Butler, D. & Pirovano, W. Scaffolding pre-assembled contigs using SSPACE. *Bioinformatics* **27**, 578-579 (2011).

8 Langmead, B., Trapnell, C., Pop, M. & Salzberg, S. L. Ultrafast and memory-efficient alignment of short DNA sequences to the human genome. *Genome Biol* **10**, R25 (2009).

9 Boetzer, M. & Pirovano, W. Toward almost closed genomes with GapFiller. *Genome Biol* **13** (2012).

10 Kent, W. J. BLAT - The BLAST-like alignment tool. *Genome Res* **12**, 656-664 (2002).

11 Parra, G., Bradnam, K. & Korf, I. CEGMA: a pipeline to accurately annotate core genes in eukaryotic genornes. *Bioinformatics* **23**, 1061-1067 (2007).

12 Haas, B. J. *et al.* De novo transcript sequence reconstruction from RNA-seq using the Trinity platform for reference generation and analysis. *Nat Protoc* **8**, 1494-1512 (2013).

13 Benson, G. Tandem repeats finder: a program to analyze DNA sequences. *Nucleic Acids Research* **27**, 573-580 (1999).

14 Xu, Z. & Wang, H. LTR_FINDER: an efficient tool for the prediction of full-length LTR retrotransposons. *Nucleic Acids Res* **35**, W265-W268 (2007).

15 Lowe, T. M. & Eddy, S. R. tRNAscan-SE: A program for improved detection of transfer RNA genes in genomic sequence. *Nucleic Acids Research* **25**, 955-964 (1997).

16 Griffiths - Jones, S. Annotating Non - Coding RNAs with Rfam. *Current protocols in bioinformatics*, doi: 10.1002/0471250953.bi1205s9 (2005).

17 Hofacker, I. L. *et al.* Fast folding and comparison of RNA secondary structures. *Monatshefte für Chemie/Chemical Monthly* **125**, 167-188 (1994).

18 Langmead, B. & Salzberg, S. L. Fast gapped-read alignment with Bowtie 2. *Nat Methods* **9**, 357-359 (2012).

19 DePristo, M. A. *et al.* A framework for variation discovery and genotyping using next-generation DNA sequencing data. *Nature Genetics* **43**, 491-498 (2011).

20 Conesa, A. *et al.* Blast2GO: a universal tool for annotation, visualization and analysis in functional genomics research. *Bioinformatics* **21**, 3674-3676 (2005).

21 Kim, D. *et al.* TopHat2: accurate alignment of transcriptomes in the presence of insertions, deletions and gene fusions. *Genome Biol* **14**, R36 (2013).

22 Trapnell, C. *et al.* Differential gene and transcript expression analysis of RNA-seq experiments with TopHat and Cufflinks. *Nat Protoc* **7**, 562-578 (2012).

23 Haas, B. J. *et al.* Automated eukaryotic gene structure annotation using EVidenceModeler and the program to assemble spliced alignments. *Genome Biol* **9**, R7 (2008).

24 Iwata, H. & Gotoh, O. Benchmarking spliced alignment programs including Spaln2, an extended version of Spaln that incorporates additional species-specific features. *Nucleic Acids Research* **40**, e161 (2012).

25 Stanke, M., Diekhans, M., Baertsch, R. & Haussler, D. Using native and syntenically mapped cDNA alignments to improve de novo gene finding. *Bioinformatics* **24**, 637-644 (2008).

26 Lukashin, A. V. & Borodovsky, M. GeneMark.hmm: new solutions for gene finding. *Nucleic Acids Research* **26**, 1107-1115 (1998).

27 Pertea, M. & Salzberg, S. L. Using GlimmerM to find genes in eukaryotic genomes. *Current protocols in bioinformatics*, doi: 10.1002/0471250953.bi0404s00 (2002).

28 Korf, I. Gene finding in novel genomes. *Bmc Bioinformatics* **5**, doi: 10.1186/1471-2105-5-59 (2004).

29 Jones, P. *et al.* InterProScan 5: genome-scale protein function classification. *Bioinformatics* **30**, 1236-1240 (2014).

30 Hahn, C., Fromm, B. & Bachmann, L. Comparative Genomics of Flatworms (Platyhelminthes) Reveals Shared Genomic Features of Ecto- and Endoparastic Neodermata. *Genome Biology and Evolution* **6**, 1105-1117 (2014).

31 Amit, M. *et al.* Differential GC Content between Exons and Introns Establishes Distinct Strategies of Splice-Site Recognition. *Cell Reports* **1**, 543-556 (2012).

32 Li, L., Stoeckert, C. J. & Roos, D. S. OrthoMCL: Identification of ortholog groups for eukaryotic genomes. *Genome Res* **13**, 2178-2189 (2003).

33 Yang, Z. H. PAML: a program package for phylogenetic analysis by maximum likelihood. *Computer Applications in the Biosciences* **13**, 555-556 (1997).

34 Kurtz, S. *et al.* Versatile and open software for comparing large genomes. *Genome Biol* **5**, R12 (2004).

35 Wang, Y. P. *et al.* MCScanX: a toolkit for detection and evolutionary analysis of gene synteny and collinearity. *Nucleic Acids Research* **40**, e49 (2012).

36 Salichos, L. & Rokas, A. Evaluating Ortholog Prediction Algorithms in a Yeast Model Clade. *Plos One* **6**, e18755 (2011).

37 Larkin, M. A. *et al.* Clustal W and clustal X version 2.0. *Bioinformatics* **23**, 2947-2948 (2007).

38 Capella-Gutierrez, S., Silla-Martinez, J. M. & Gabaldon, T. trimAl: a tool for automated alignment trimming in large-scale phylogenetic analyses. *Bioinformatics* **25**, 1972-1973 (2009).

39 Darriba, D., Taboada, G. L., Doallo, R. & Posada, D. ProtTest 3: fast selection of best-fit models of protein evolution. *Bioinformatics* **27**, 1164-1165 (2011).

40 Posada, D. ModelTest Server: a web-based tool for the statistical selection of models of nucleotide substitution online. *Nucleic Acids Res* **34**, W700-W703 (2006).

41 Stamatakis, A. RAxML-VI-HPC: Maximum likelihood-based phylogenetic analyses with thousands of taxa and mixed models. *Bioinformatics* **22**, 2688-2690 (2006).

42 Felsenstein, J. PHYLIP: Phylogenetic inference program, version 3.6. *University of Washington, Seattle* (2005).

43 De Bie, T., Cristianini, N., Demuth, J. P. & Hahn, M. W. CAFE: a computational tool for the study of gene family evolution. *Bioinformatics* **22**, 1269-1271 (2006).

44 Bouckaert, R. *et al.* BEAST 2: A Software Platform for Bayesian Evolutionary Analysis. *Plos Computational Biology* **10**, e1003537 (2014).

45 Douzery, E. J. P., Snell, E. A., Bapteste, E., Delsuc, F. & Philippe, H. The timing of eukaryotic evolution: Does a relaxed molecular clock reconcile proteins and fossils? *Proceedings of the National Academy of Sciences of the United States of America* **101**, 15386-15391 (2004).

46 Parfrey, L. W., Lahr, D. J. G., Knoll, A. H. & Katz, L. A. Estimating the timing of early eukaryotic diversification with multigene molecular clocks. *Proceedings of the National Academy of Sciences of the United States of America* **108**, 13624-13629 (2011).

47 Xia, X. & Xie, Z. DAMBE: Software package for data analysis in molecular biology and evolution. *J Hered* **92**, 371-373 (2001).

48 Enright, A. J., Van Dongen, S. & Ouzounis, C. A. An efficient algorithm for large-scale detection of protein families. *Nucleic Acids Research* **30**, 1575-1584 (2002).

49 Maere, S. *et al.* Modeling gene and genome duplications in eukaryotes. *P Natl Acad Sci USA* **102**, 5454-5459 (2005).

50 Vanneste, K., Van de Peer, Y. & Maere, S. Inference of Genome Duplications from Age Distributions Revisited. *Mol Biol Evol* **30**, 177-190 (2013).

51 Katoh, K. & Standley, D. M. MAFFT Multiple Sequence Alignment Software Version 7: Improvements in Performance and Usability. *Molecular Biology and Evolution* **30**, 772-780 (2013).

52 Zhang, Z. *et al.* ParaAT: A parallel tool for constructing multiple protein-coding DNA alignments. *Biochem Bioph Res Co* **419**, 779-781 (2012).

53 Nystedt, B. *et al.* The Norway spruce genome sequence and conifer genome evolution. *Nature* **497**, 579-584 (2013).

54 O'Brien, K. P., Remm, M. & Sonnhammer, E. L. L. Inparanoid: a comprehensive database of eukaryotic orthologs. *Nucleic Acids Research* **33**, D476-D480 (2005).

55 Lynch, M. & Conery, J. S. The evolutionary fate and consequences of duplicate genes. *Science* **290**, 1151-1155 (2000).

56 Lynch, M. & Conery, J. S. The evolutionary demography of duplicate genes. *Journal of structural and functional genomics* **3**, 35-44 (2003).

57 Neafsey, D. E. *et al.* Mosquito genomics. Highly evolvable malaria vectors: the genomes of 16 Anopheles mosquitoes. *Science* **347**, 1258522 (2015).

58 Alexeyenko, A., Tamas, I., Liu, G. & Sonnhammer, E. L. L. Automatic clustering of orthologs and inparalogs shared by multiple proteomes. *Bioinformatics* **22**, E9-E15 (2006).

59 Benjamini, Y. & Hochberg, Y. Controlling the False Discovery Rate - a Practical and Powerful Approach to Multiple Testing. *J Roy Stat Soc B Met* **57**, 289-300 (1995).

60 Krogh, A., Larsson, B., von Heijne, G. & Sonnhammer, E. L. L. Predicting transmembrane protein topology with a hidden Markov model: Application to complete genomes. *Journal of Molecular Biology* **305**, 567-580 (2001).

61 Kall, L., Krogh, A. & Sonnhammer, E. L. L. Advantages of combined transmembrane topology and signal peptide prediction - the Phobius web server. *Nucleic Acids Research* **35**, W429-W432 (2007).

62 Petersen, T. N., Brunak, S., von Heijne, G. & Nielsen, H. SignalP 4.0: discriminating signal peptides from transmembrane regions. *Nature Methods* **8**, 785-786 (2011).

63 Bendtsen, J. D., Jensen, L. J., Blom, N., von Heijne, G. & Brunak, S. Feature-based prediction of non-classical and leaderless protein secretion. *Protein Engineering Design & Selection* **17**, 349-356 (2004).

64 Emanuelsson, O., Brunak, S., von Heijne, G. & Nielsen, H. Locating proteins in the cell using TargetP, SignalP and related tools. *Nat Protoc* **2**, 953-971 (2007).

65 de Castro, E. *et al.* ScanProsite: detection of PROSITE signature matches and ProRule-associated functional and structural residues in proteins. *Nucleic Acids Research* **34**, W362-W365 (2006).

66 Pierleoni, A., Martelli, P. L. & Casadio, R. PredGPI: a GPI-anchor predictor. *Bmc Bioinformatics* **9**, 392

(2008).

67 Rawlings, N. D. & Morton, F. R. The MEROPS batch BLAST: A tool to detect peptidases and their non-peptidase homologues in a genome. *Biochimie* **90**, 243-259 (2008).

68 Becker, M. M. *et al.* Cloning and characterization of the Schistosoma japonicum aspartic proteinase involved in hemoglobin degradation (vol 270, pg 24496, 1995). *J Biol Chem* **272**, 17246-17246 (1997).

69 Eddy, S. R. Accelerated Profile HMM Searches. *Plos Comput Biol* **7**, e1002195 (2011).

70 Berriman, M. *et al.* The genome of the blood fluke Schistosoma mansoni. *Nature* **460**, 352-358 (2009).
